# Peptides targeting RAB11A–FIP2 complex inhibit HPIV3, RSV, and IAV replication as broad-spectrum antivirals

**DOI:** 10.1186/s13578-025-01384-z

**Published:** 2025-04-21

**Authors:** Yanliang Jiang, Yongliang Zhao, Jie Deng, Xiaoyan Wu, Jian Li, Dong Guo, Ke Xu, Yali Qin, Mingzhou Chen

**Affiliations:** 1 State Key Laboratory of Virology and Biosafety, Hubei Provincial Research Center for Basic Biological Sciences, College of Life Sciences, Wuhan University, Wuhan, 430072 China; 2 Shool of Life Sciences, Hubei University, Wuhan, 430062 China; 3 Hubei Jiangxia Laboratory, Wuhan, 430200 China; 4 Taikang Center for Life and Medical Sciences, Wuhan University, Wuhan, 430072 China; 5 National “111” Center for Cellular Regulation and Molecular Pharmaceutics, Key Laboratory of Fermentation Engineering (Ministry of Education), Hubei University of Technology, Wuhan, China

**Keywords:** HPIV3, RSV, IAV, RAB11A, YT-DRI, Cytoskeletal framework, Broad-spectrum antivirals

## Abstract

**Background:**

The cytoskeletal framework plays a critical role in the early stages of human parainfluenza virus type 3 (HPIV3) replication, including viral mRNA synthesis and translation. However, its contribution to later stages of infection, particularly in the context of RNA biology, is not well understood. This study focuses on the role of the cytoskeleton in viral nucleocapsid (vRNP, a ribonucleoprotein complex essential for RNA virus replication) transport, assembly, and budding, and explores the cooperative role of the small GTPase RAB11A and its effector RAB11 family interacting protein 2 (FIP2) in vRNP trafficking. These processes are crucial for respiratory RNA viruses like respiratory syncytial virus (RSV) and influenza A virus (IAV), highlighting the importance of RNA–protein interactions in viral pathogenesis.

**Results:**

Through the use of cytoskeleton-depolymerizing agents, the study identified actin microfilaments as indispensable for vRNP transport, viral assembly, and viral particle budding. It also revealed the importance of the RAB11A–FIP2 complex in these processes, which are critical for the intracellular trafficking of viral RNA. The development of peptides targeting the RAB11A–FIP2 complex led to the suppression of RAB11A function in infected cells, resulting in vRNP aggregation in the cytoplasm and reduced viral replication. The peptide YT-DRI showed strong broad-spectrum antiviral activity against HPIV3, RSV, and IAV in cellular and animal models and was effective against co-infections in vitro. The antiviral effects of YT-DRI were abolished upon deletion of RAB11A or core components of the RAB11A pathway.

**Conclusion:**

This work introduces a promising broad-spectrum antiviral strategy for respiratory tract infections by targeting the RAB11A–FIP2 complex, which regulates the transport and assembly of viral RNA. By disrupting this pathway, YT-DRI effectively inhibits the replication of multiple respiratory RNA viruses, including HPIV3, RSV, and IAV.

**Supplementary Information:**

The online version contains supplementary material available at 10.1186/s13578-025-01384-z.

## Introduction

RNA viruses are major pathogens responsible for respiratory tract diseases, including respiratory syncytial virus (RSV) [1], influenza A virus (IAV), metapneumovirus, rhinovirus, coronavirus, and human parainfluenza viruses (HPIVs). Among the four HPIVs serotypes, HPIV3 is the most prevalent and causes severe respiratory tract infections in infants, immunocompromised individuals, and the elderly [2, 3]. Despite its clinical significance, there are currently no vaccines or antiviral drugs available for HPIVs infections [4–7].

Respiratory viral co-infections occur in 3–30% of cases, imposing a substantial economic burden on individuals, families, and healthcare systems [8–11]. While existing vaccines and antivirals target individual viruses, broad-spectrum antivirals (BSAs) offer the potential to inhibit multiple viruses by targeting shared host pathways. Two primary strategies for BSAs include nucleoside/nucleotide analogs and host-targeting antivirals (HTAs) [12]. However, the former class often face limitations such as drug resistance and toxicity, making HTAs an increasingly attractive option [13, 14].

HPIV3, a member of the *Paramyxoviridae* family, shares structural similarities with RSV and possesses a negative-sense, single-stranded RNA genome. Its genome encodes six structural proteins, including the nucleoprotein (N), phosphoprotein (P), RNA-dependent RNA polymerase (L), matrix protein (M), and two glycoproteins—hemagglutinin/neuraminidase (HN) and the fusion protein (F). The viral RNA, encapsulated by N, P, and L, forms the ribonucleoprotein (RNP) complex essential for viral RNA synthesis. The M protein plays a crucial role in the budding, as its expression alone is sufficient to initiate the release of virus-like particles (VLPs) [15, 16]. While previous studies have focused on early stages of HPIV3 replication, such as viral mRNA synthesis and translation [17–23], our study expands the understanding of vRNP trafficking, assembly, and budding. We demonstrate that microtubules and actin filaments play distinct roles in late-stage replication, with actin having a more pronounced effect.

Unlike many enveloped viruses, HPIV3 employs an endosomal sorting complex required for transport (ESCRT)-independent budding mechanism. Instead, small GTPases, particularly RAB GTPase, regulate vesicular transport and viral replication [24–28]. RAB11, a key member of the RAB GTPase family, interacts with RAB11-family-interacting proteins (FIPs) to facilitate vesicular trafficking [29–34]. The RAB binding domains (RBDs), located at the carboxyl termini of RAB11-FIPs, are conserved across the RAB-FIP family and interact specifically with the active form of RAB11 [35, 36]. The RAB11-FIP complex is regulated by GTP/GDP binding, with mutations such as Q70L (GTP-bound) and S25N (GDP-bound) stabilizing active and states, respectively. RAB11 is implicated in the replication of multiple viruses, including RSV and IAV, which often co-circulate and cause significant clinical disease [37–41]. In the case of HPIV1, suppressing RAB11 expression leads to vRNP aggregation in the cytoplasm and reduced virion formation [42]; although the precise mechanism remains unclear. Collectively, these findings suggest that RAB11 may serve as a common target for broad-spectrum antiviral strategies.

In this study, we highlight the critical role of actin microfilaments in HPIV3 vRNPs transport, assembly, and budding, processes regulated by the RAB11A–FIP2 complex. We designed a series of peptides targeting this complex, among which YT-DRI exhibited potent antiviral activity against HPIV3 in a RAB11A-dependent manner. Notably, YT-DRI also demonstrated broad-spectrum antiviral effects against RSV and IAV in vitro and in vivo, and effectively suppressed co-infections by HPIV3, RSV, and IAV. Collectively, our findings underscore the potential of targeting the RAB11A–FIP2 complex as a strategy for developing broad-spectrum antivirals against respiratory RNA viruses.

## Results

### An actin-dependent mechanism orchestrates the late stages of HPIV3 infection

Previous studies have highlighted the critical role of drugs that influence cytoskeletal function during the early stages of HPIV3 replication. Building upon this foundation, we investigated the effects of microtubule and actin inhibitors, specifically Nocodazole (NCZ) and Cytochalasin D (Cyto D), on the later stages of the HPIV3 life cycle.

**Fig. 1:**
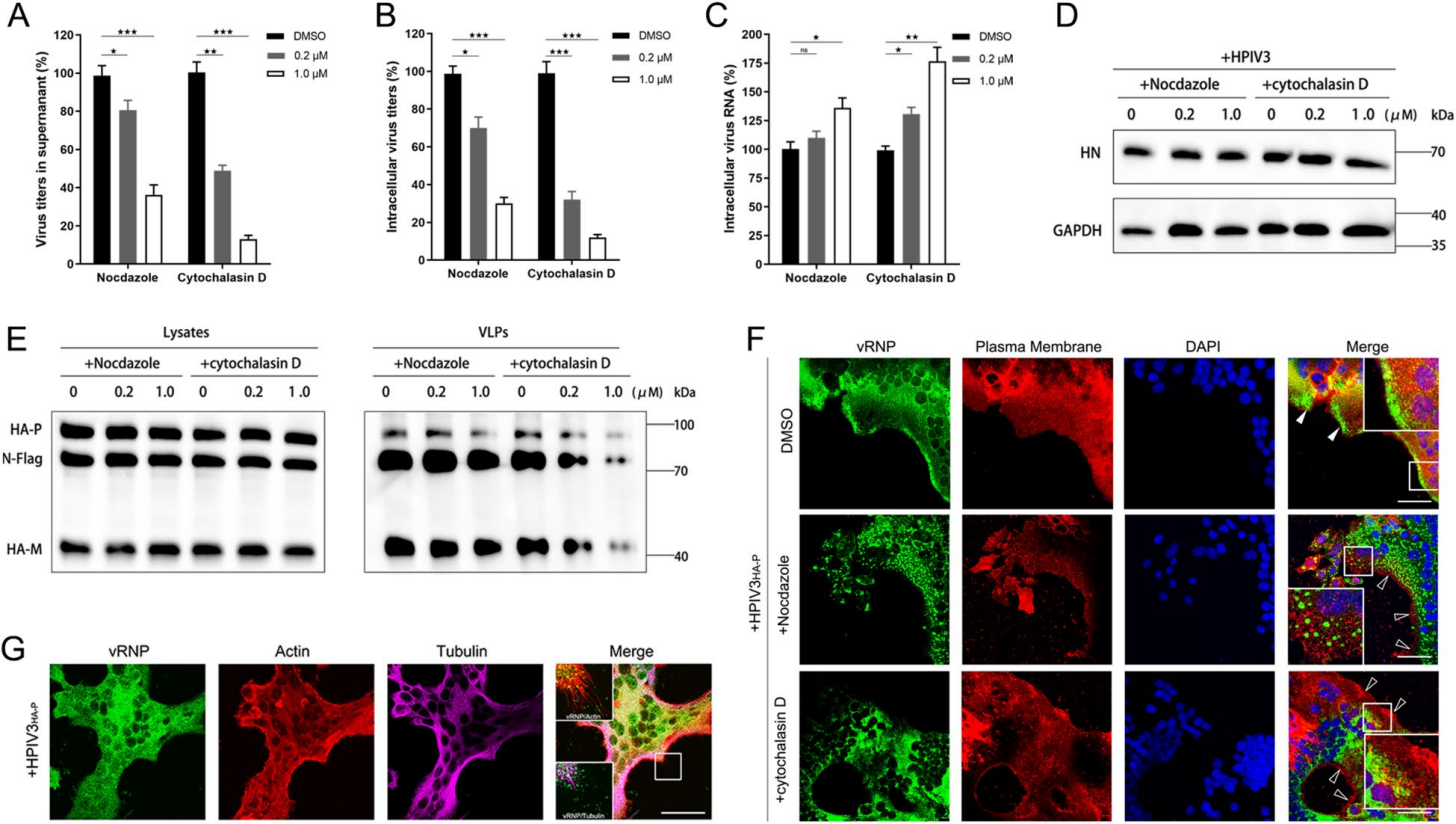
Actin plays a critical role in the late stages of HPIV3 infection. **A**–**D** Impact of cytoskeleton inhibitors on HPIV3 replication. HeLa cells infected with HPIV3 for 18 h were treated with Nocodazole (NCZ) or Cytochalasin D (Cyto D). At 24 h post infection (hpi), the virions released in the cell supernatant (**A**) and the quantity of intracellular progeny virus particles (**B**) were determined by TCID_50_ assays. Cells were collected for RT-qPCR analysis of intracellular viral RNA (**C**), and Western Blot (WB) analysis of intracellular viral protein (**D**). **E** Influence of cytoskeleton inhibitors on HPIV3 VLPs production. 293T cells transfected with indicated plasmids for 30 h were treated with NCZ or Cyto D for 6 h. Cell lysates and VLPs were prepared and analyzed by WB. **F** Impact of cytoskeleton inhibitors on vRNP distribution. HeLa cells infected with HPIV3_HA-P_ (MOI = 0.01) for 18 h were treated with NCZ or Cyto D for 6 h. Fluorescent microscopy was employed to examine vRNP distribution using an anti-HA antibody (green), plasma membrane stained by Dil (red), and nuclei were stained with DAPI. Arrowheads indicate plasma membrane. Scale bar, 50 μm. **G** Progeny virions released via actin filaments. HPIV3_HA-P_-infected HeLa cells were immunofluorescently labeled for vRNP (green), actin (red), and tubulin (magenta). Scale bar, 50 μm. All experiments were independently repeated at least twice with reproducible results. Statistical significance (*p < 0.05, **p < 0.01, ***p < 0.001) was determined by two-sided unpaired t test. Data are means ± SEM

To determine the specificity and effects of these inhibitors, HeLa cells were treated with 0.2 µM or 1 µM concentrations for 6 h. Immunofluorescence assays for F-actin and microtubule visualization demonstrated that Cyto D selectively disrupted the structure of F-actin fibers while leaving microtubules intact (Fig. S1A). Conversely, NCZ disrupted the microtubule network without affecting F-actin structure (Fig. S1A). To assess the potential cytotoxicity of these inhibitors, cytotoxicity assays were conducted. Following treatment with the specified concentrations of NCZ or Cyto D for 6 h, cell viability was evaluated using CCK-8 assays, revealing no significant cytotoxic effects from either NCZ or Cyto D treatment (Fig. S1, B and C). To investigate the role of the cytoskeleton in the later stages of infection, HeLa cells were infected with HPIV3 for 18 h before being treated with the inhibitors for an additional 6 h. Viral titer assessments indicated that treatment with NCZ and Cyto D resulted in a significant reduction in the presence of HPIV3 virions in the supernatant (Fig. 1A) as well as a decrease in intracellular progeny virus particles, suggesting a failure in viral assembly (Fig. 1B). Notably, viral RNA synthesis and protein expression levels were unaffected by treatment with NCZ and Cyto D (Fig. 1C and D). Interestingly, in cells treated with 1 µM NCZ or Cyto D, the abundance of intracellular viral RNA appeared elevated compared to control cells, likely due to an accumulation of viral RNA resulting from the inability to release progeny virus particles into the supernatant (Fig. 1C). These findings suggest a synergistic role for actin and microtubules in the assembly and budding processes of HPIV3 infection, with actin exhibiting a more significant influence. To further confirm these observations, we analyzed the assembly and budding of HPIV3 using a virus-like particles (VLPs) assay. Plasmids encoding the N, P, and M were transfected into 293T cells, and the resulting VLPs were assessed via Western blotting. The results indicated that Cyto D had a substantial impact on VLP production, in contrast to the modest effect observed with NCZ (Fig. 1E). To investigate the role of actin in the cytoplasmic trafficking of vRNPs, we employed a recombinant HPIV3 with an HA tag fused to the N-terminus of P (HPIV3_HA-P_). Immunostaining with an anti-HA monoclonal antibody revealed that, under normal conditions, vRNPs were diffusely distributed throughout the cytoplasm, with some localization near the plasma membrane (PM) (Fig. 1F, white arrowheads). Both inhibitors altered the distribution of vRNPs, with Cyto D causing larger aggregates in the cytoplasm that hindered their trafficking toward the PM (Fig. 1F, hollow arrowheads). High-magnification images illustrated a significant difference in the co-localization of actin and microtubules with progeny virions (Fig. 1G). These findings highlight the essential contributions of both actin and microtubules in viral assembly and release, with actin filaments exhibiting a more significant role on the transport of vRNPs and the release of viral particles.

### RAB11A regulates late-stage processes in HPIV3: from transport to budding

**Fig. 2:**
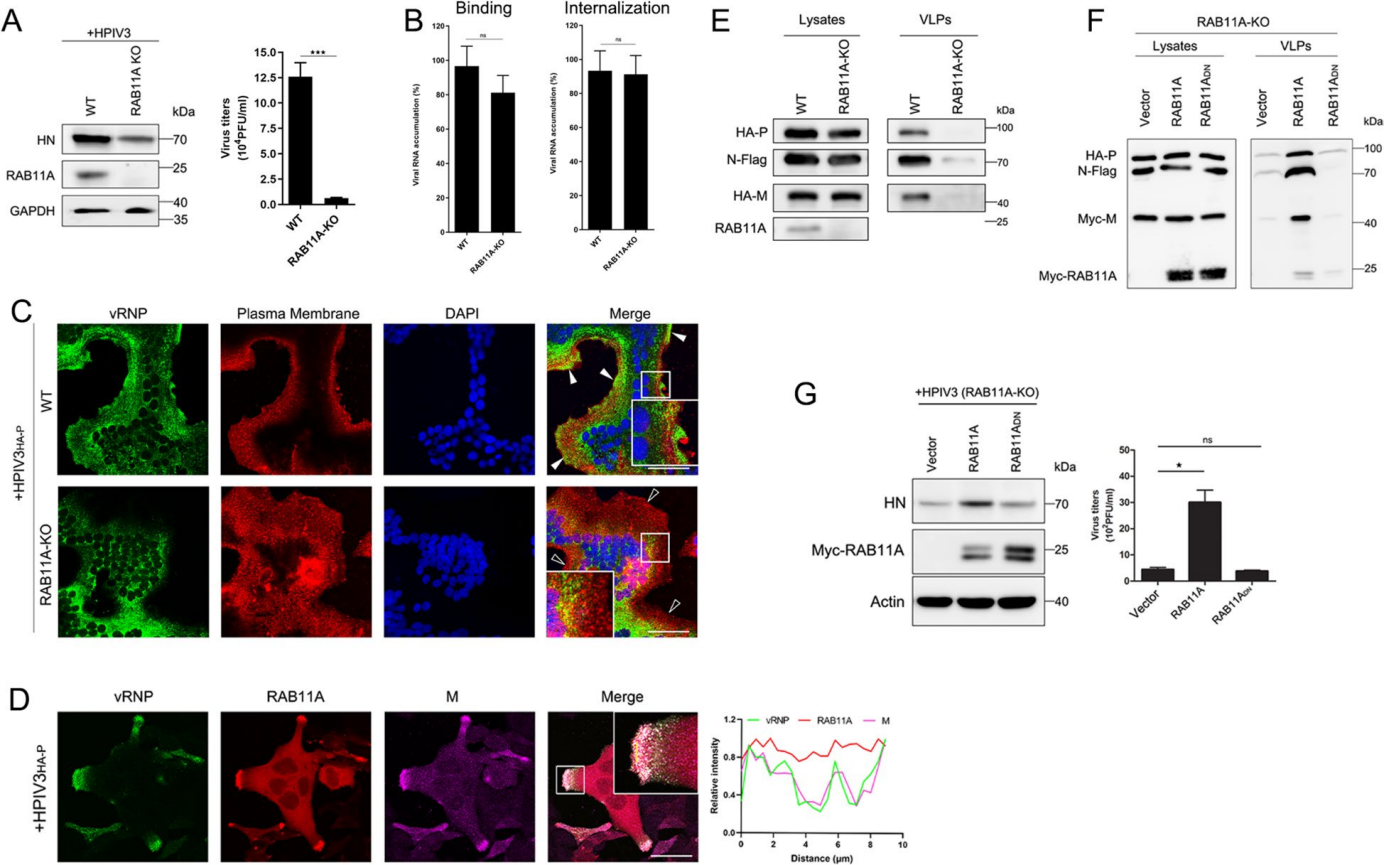
A RAB11A-dependent mechanism governs the progresses of HPIV3 infection: from vRNP transport to budding. **A** Impact of RAB11A depletion on HPIV3 replication in HeLa cells. WT and RAB11A-depleted HeLa cells infected with HPIV3 (MOI = 0.01) for 24 h were analyzed for viral protein by WB, and viral titers in the cell supernatant were determined by TCID_50_. **B** RAB11A is not required for HPIV3 binding and internalization. Analysis of the virions binding and internalized into HeLa of HPIV3 to WT and RAB11A-depleted HeLa cells using RT-qPCR. Cells were incubated with the virus at an MOI of 5 for 1 h at 4 °C, and then washed 3 times with ice-cold PBS to remove unbound viral particles, and then RNA could be extracted for binding analysis. For internalization assay, fresh complete DMEM was added following binding processes, and the cells were incubated at 37 °C for another 1 h to allow virus internalization. HPIV3 RNA copy numbers were normalized to the mean values for WT cells in each assay. **C** Disruption of vRNP trafficking due to RAB11A depletion. WT and RAB11A-depleted HeLa cells infected with HPIV3_HA-P_ (MOI = 0.01) for 24 h were immunofluorescently labeled for vRNP (green), plasma membranes stained by Dil (red), and with nuclei stained by DAPI. Arrowheads indicate plasma membrane. Scale bar, 50 μm. **D** Co-localization of vRNP, RAB11A, and M protein at the plasma membrane. HeLa cells transfected with plasmids expressing RAB11A and M protein were infected with HPIV3_HA-P_ (MOI = 0.01) for 24 h and immunofluorescently labeled for vRNP (green), RAB11A (red), M (magenta), with nuclei stained by DAPI. Scale bar, 50 μm. Histogram indicates fluorescence intensities in three channels along the yellow line. **E** Disruption of VLPs production due to RAB11A depletion. WT and RAB11A-depleted 293 T cells transfected with indicated plasmids for 48 h had cell lysates and VLPs prepared and analyzed by WB. **F** Restoration of VLPs production by active RAB11A in RAB11A-KO cells. RAB11A-depleted 293 T cells transfected with plasmids encoding N, P, and M protein, along with RAB11A or RAB11A_DN_, were analyzed for cell lysates and VLPs by WB at 48 h post transfection. **G** Restoration of HPIV3 replication by active RAB11A in RAB11A-depleted cells. RAB11A-depleted HeLa cells transfected with plasmids encoding RAB11A or RAB11A_DN_ were infected with HPIV3 (MOI = 0.01) for 24 h. Viral protein was analyzed by WB, and viral titers in the cell supernatant were determined by TCID_50_. All experiments were independently repeated at least twice with reproducible results. Statistical significance (*p < 0.05, **p < 0.01, ***p < 0.001)  was determined by two-sided unpaired t test. Data are means ± SEM

Our study highlights the indispensable role of actin filaments in the late stages of HPIV3 infection, particularly in the transport of vRNPs, a process previously established to be independent of ESCRT mechanisms [15]. Expanding our investigation to other vesicular transport regulators, specifically RAB GTPases, we examined their involvement in the latter stages of the HPIV3 life cycle. Co-localization studies with various constitutively active RAB proteins revealed a distinctive association between vRNPs and the constitutively active form of RAB11A (RAB11A_CA_) (Fig. S2). This specificity suggests that RAB11A plays a unique role in vRNP cytoplasmic trafficking within the RAB GTPase family. To assess the essentiality of RAB11A in viral replication, we generated a RAB11A knockout (KO) HeLa cell line using CRISPR-Cas9 technology. Compared to wild-type (WT) HeLa cells, RAB11A-KO cells displayed a significant reduction in infectious particle production at 24 h post-infection (hpi) (Fig. 2A). To elucidate how RAB11A regulates HPIV3 replication, we first compared the viral binding and entry capabilities in WT and RAB11A-KO cells, finding that the knockout of RAB11A did not affect viral binding or entry (Fig. 2B). Immunostaining of HPIV3_HA-P_-infected cells demonstrated that RAB11A deficiency adversely affected vRNP distribution, suppressing the formation of vRNP clusters at the plasma membrane (PM) while leading to large aggregates in the cytoplasm (Fig. 2C). This indicates that RAB11A plays a critical role in vRNP trafficking to the PM, where the transport of progeny vRNPs from the cytoplasm to sites of viral assembly is essential for efficient production of infectious particles. To further investigate the involvement of RAB11A in assembly and budding processes—primarily driven by the M protein—we employed fluorescence microscopy. Co-expression of RAB11A and M proteins in HPIV3_HA-P_-infected HeLa cells revealed efficient co-localization of vRNPs and M proteins at the PM, underscoring RAB11A’s crucial role in vRNP transport toward the assembly site (Fig. 2D). In assessing VLP budding, we observed a significant decrease in VLP production upon RAB11A depletion in 293T cells (Fig. 2E). To dissect the mechanism underlying RAB11A-mediated VLP release, we evaluated the impact of wild-type and dominant-negative (DN) mutant RAB11A proteins in RAB11A-KO 293T cells. VLP production was restored only in cells reintroduced with active RAB11A, not with the inactive RAB11A_DN_, highlighting the necessity of the functional GTP/GDP RAB11A cycle (Fig. 2F). Further validating the importance of GTP-bound RAB11A in HPIV3 infection, RAB11A-KO HeLa cells transfected with plasmids expressing RAB11A and RAB11A_DN_ exhibited restored infection solely with active RAB11A, consistent with the VLP assay results (Fig. 2G). Collectively, our observations indicate that an actin- and RAB11A-dependent vesicular transport pathway is pivotal for HPIV3 assembly and budding, in contrast to the minor effects observed with microtubule disruption on HPIV3 replication.

### The interaction of vRNP with RAB11A is essential for vRNP trafficking during late-stage HPIV3 processes

**Fig. 3:**
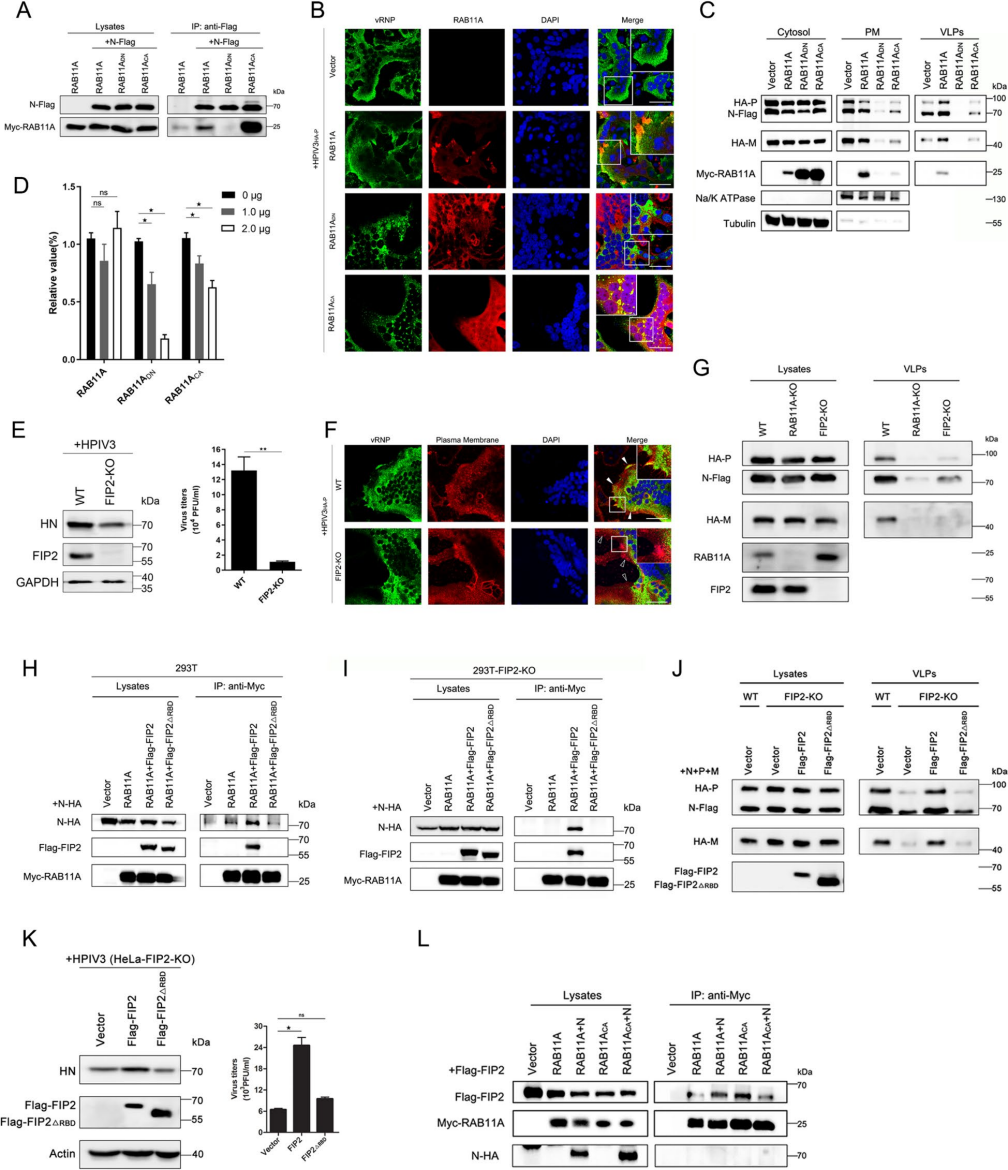
Formation of RAB11A–FIP2 complex is necessary to HPIV3 infection. **A** N protein orchestrates an exclusive interaction with active RAB11A. 293T cells, individually or jointly transfected with plasmids encoding N-Flag, Myc-RAB11A, Myc-RAB11A_DN_, or Myc-RAB11A_CA_, were subjected to co-IP assays at 36 h post-transfection. Immunoprecipitation with anti-Flag magnetic beads followed by WB revealed the specific interaction. **B** Diverse RAB11A forms and their co-localization with vRNP. HeLa cells expressing RAB11A, RAB11A_DN_, or RAB11A_CA_ were infected with HPIV3_HA-P_ (MOI = 0.01) for 24 h. Immunofluorescent detection of vRNP (green) and RAB11A (red) showcased distinct co-localization patterns. Scale bar, 50 μm. **C** Impact of RAB11A variants on vRNP distribution and VLPs production. 293T cells transfected with RAB11A variants were fractionated into cytosol and plasma membrane (PM) fractions. WB analysis of vRNP and VLPs in different cellular compartments demonstrated the influence of RAB11A forms. Tubulin and Na/K ATPase served as cytosol or PM markers. **D** RAB11A_DN_ and RAB11A_CA_ act as suppressors of HPIV3 replication. Hela cells overexpressing RAB11A, RAB11A_DN_, or RAB11A_CA_ were infected with HPIV3(MOI = 0.01) for 24 h, and viral titers in the supernatant were quantified by TCID_50_. **E**, **F** FIP2 depletion impedes HPIV3 replication and disrupts vRNP trafficking. WT and FIP2-depleted HeLa cells, infected with HPIV3(MOI = 0.01) (**E**) or HPIV3_HA-P_ (MOI = 0.01) (**F**) for 24 h, exhibited reduced viral protein levels and impaired vRNP trafficking, as evidenced by immunofluorescent detection of vRNP (green), plasma membrane stained with Dil (red), and nuclei stained with DAPI. Scale bar, 50 μm. **G** FIP2 depletion hampers VLPs production. 293T cells, depleted of RAB11A or FIP2, were transfected with plasmids for 48 h. WB analysis of cell lysates and VLPs demonstrated the impact of FIP2 on VLP production. **H** and **I** N protein-RAB11A interaction in the presence of different FIP2 forms. WT or FIP2-depleted 293T cells were transfected with plasmids, and co-IP assays revealed the dynamic interaction between N protein and RAB11A influenced by various FIP2 forms. **J** FIP2 restoration of VLPs production in FIP2-depleted cells. FIP2-depleted 293T cells, transfected with plasmids, exhibited restored VLPs production as seen in WB analysis. **K** FIP2 restoration of HPIV3 replication in FIP2-depleted cells. FIP2-KO Hela cells, transfected with plasmids, displayed revived HPIV3 replication, evidenced by viral protein levels (WB) and TCID_50_. **L** The N protein competes with FIP2 for binding to RAB11A_CA_. 293T cells co-transfected with plasmids revealed the competitive binding between N protein and FIP2 for RAB11A_CA_, elucidated through co-IP assays and subsequent WB analysis. All experiments were independently replicated at least twice with consistent results. Statistical significance (*p < 0.05, **p < 0.01) was determined by two-sided unpaired t-test. Data are presented as means ± SEM

To elucidate the interaction between vRNPs and RAB11A at the cell periphery, we employed co-immunoprecipitation (co-IP) assays using a plasmid encoding Flag-tagged RAB11A. Co-expressing Flag-RAB11A with the N or P of HPIV3, we found that only the N protein co-precipitated with Flag-RAB11A (Fig. S3A). This interaction suggests a potential association between vRNPs and RAB11A, likely mediated by the N protein. To further dissect this interaction, we investigated the relationship between the N protein and RAB11A using co-IP assays with three variants of Myc-RAB11A as precipitants. The constitutively active form of RAB11A (RAB11A_CA_), previously shown to co-localize with vRNPs (Fig. S2), served as a positive control. The results demonstrated a specific interaction between the N protein and active RAB11A, particularly with RAB11A_CA_ (Fig. 3A). This finding is consistent with microscopy data that highlighted co-localization of vRNPs with RAB11A and RAB11A_CA_, whereas no such interaction was observed with RAB11A_DN_ (Fig. 3B). Interestingly, while RAB11A expression did not alter the overall distribution of vRNPs, it did co-localize with vRNPs at the PM. However, excessive interaction between RAB11A_CA_ and the N protein resulted in the formation of vRNP aggregates, hindering their migration to the PM. This suggests that overexpression of RAB11A_CA_ may impair the recycling capacity of infected cells, potentially limiting vRNP accumulation at the PM essential for virion formation. To investigate the implications for viral budding, we assessed vRNP levels in the PM fraction. After transfecting 293T cells with N, P, and M proteins alongside either RAB11A, RAB11A_DN_, or RAB11A_CA_, we observed reduced levels of N, P, and M proteins in both the PM fraction and VLPs when RAB11A_DN_ and RAB11A_CA_ were present, compared to RAB11A (Fig. 3C). To correlate these location changes with HPIV3 infection, we conducted an infection assay in cells expressing RAB11A, RAB11A_DN_, or RAB11A_CA_. Supernatant titers significantly decreased in cells expressing RAB11A_DN_ and RAB11A_CA_ in a dose-dependent manner, while titers in RAB11A-expressing cells remained unchanged (Fig. 3D). Collectively, these findings emphasize the necessity of a balanced interaction between the N protein and RAB11A for effective vRNP trafficking during the late stages of the HPIV3 life cycle.

### Formation of the RAB11A–FIP2 complex is a prerequisite for RAB11A-dependent vRNP trafficking

Intracellular transport of HPIV3 vRNPs to the PM may necessitate bridging proteins, such as RAB11-family interacting proteins (FIPs) [37, 43, 44], alongside actin, that work as adaptors or increase the affinity of motors for membranes. FIP2, known for mediating the trafficking of recycling endosomes through direct interaction with Myosin Vb [32], a widely expressed actin-based motor, is crucial for facilitating an actin- and RAB11A-dependent vRNP trafficking pathway. To evaluate the significance of FIP2, we investigated the impact of FIP2 KO on HPIV3 infection, revealing a marked reduction in viral replication in FIP2-KO HeLa cells compared to wild-type HeLa cells (Fig. 3E). When assessing the effect of FIP2 depletion on vRNP distribution, a dramatic decrease in vRNP association at the PM was observed in FIP2-KO cells infected with HPIV3_HA-P_ compared to wild-type HeLa cells, underscoring FIP2's essential role in vRNP trafficking to the PM (Fig. 3F). Furthermore, a VLP production assay in wild-type and FIP2-depleted 293T cells, with RAB11A-depleted cells as a control, demonstrated impaired VLP production in FIP2-depleted cells, confirming FIP2’s role in HPIV3 VLP production (Fig. 3G). In addition to bridging recycling endosomes and actin filaments, FIP2 has been implicated in a complex relationship with active RAB11A and influenza A virus (IAV) RNPs, where vRNPs can outcompete FIP2 for RAB11A binding [39, 45]. To investigate FIP2’s involvement in the interaction between HPIV3 vRNP and RAB11A, we examined the binding of the N protein to active RAB11A using co-IP assays in wild-type 293T cells. The results indicated that the N-RAB11A interaction was enhanced in the presence of FIP2, but not with the RAB11A binding domain deletion mutant of FIP2 (FIP2_△RBD_) (Fig. 3H), emphasizing FIP2's facilitative role in the N-RAB11A interaction when capable of binding RAB11A. Subsequent co-IP assays in FIP2-depleted 293T cells revealed that only the restoration of FIP2 reinstated the N-RAB11A interaction, underscoring the importance of RAB11A–FIP2 complex formation (Fig. 3I). Importantly, no direct interaction between FIP2 and the N protein was detected (Fig. S3B). To assess the role of the RAB11A–FIP2 interaction in HPIV3 infection, FIP2-KO HeLa cells were transfected with plasmids expressing FIP2 and FIP2_△RBD_, followed by HPIV3 infection. Only FIP2 restored viral infection, while FIP2_△RBD_ did not (Fig. 3K), aligning with the findings from the VLP assay (Fig. 3J). These results collectively suggest that the formation of the RAB11A–FIP2 complex is a prerequisite for RAB11A-dependent vRNP trafficking.

While our data indicate that a modest interaction between vRNP and RAB11A enhances vRNP trafficking, overexpression of RAB11A_CA_ appears to inhibit this process, warranting further investigation into the specific mechanisms involved. Given FIP2’s role in mediating the trafficking of recycling endosomes through its interaction with actin-based motors, it is plausible that vRNP competes with FIP2 for binding to RAB11A_CA_ during HPIV3 infection, potentially impeding trafficking. Co-IP assays supported this hypothesis, revealing decreased levels of FIP2 co-purifying with RAB11A_CA_ in the presence of the N protein, while no change was observed with RAB11A (Fig. 3L). These studies underscore the critical importance of the RAB11A–FIP2 complex in HPIV3 infection, suggesting that the vRNP is transported as a ternary complex of vRNP-RAB11-FIP2, rather than merely competing with FIP2 for binding to RAB11A as seen in IAV infection [39, 45].

### Targeting the RAB11A–FIP2 interaction as a strategy to inhibit RAB11A-dependent infection

**Fig. 4:**
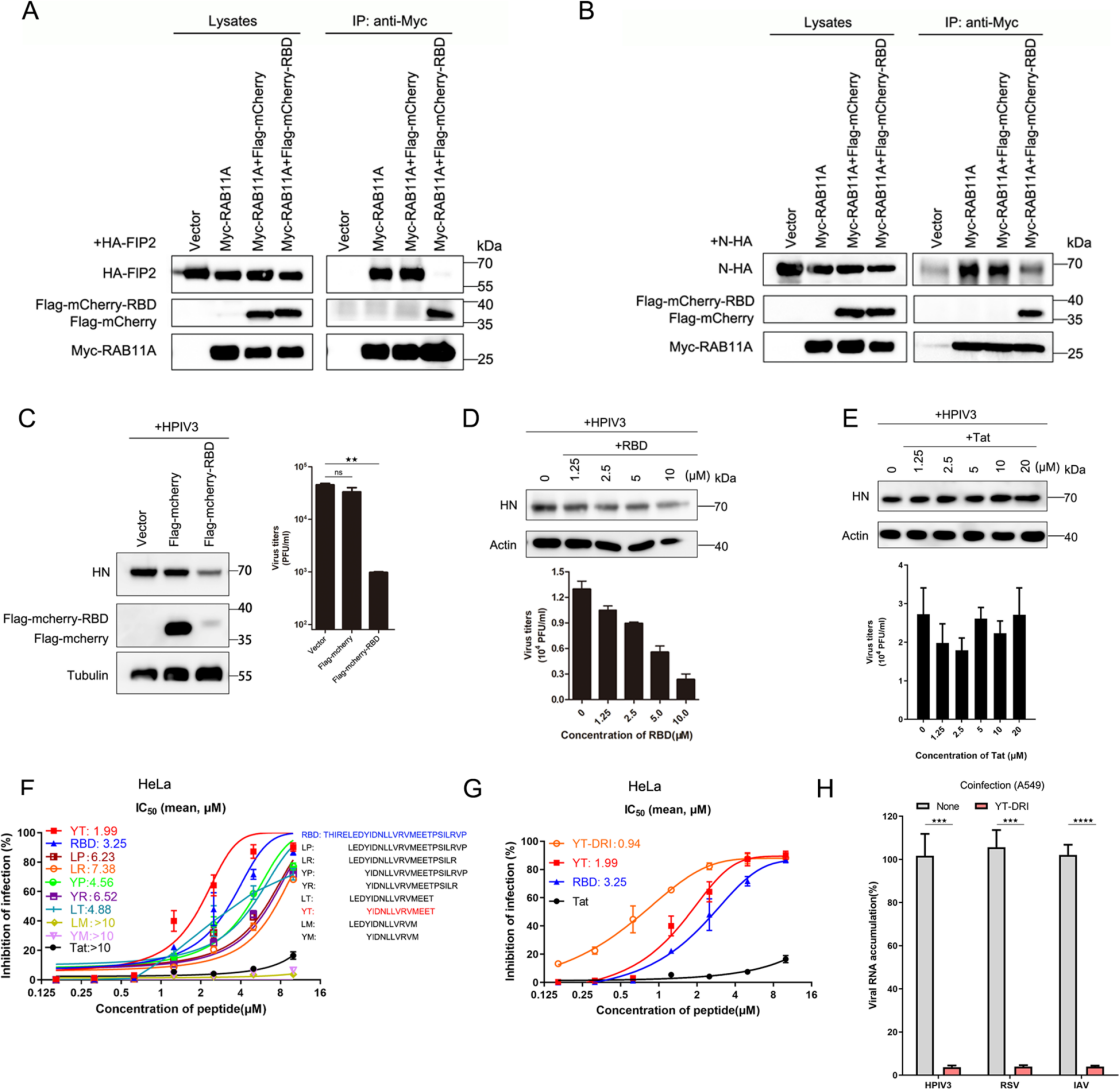
Antiviral activity of peptides targeting RAB11A–FIP2 complex against respiratory RNA viruses infection in cells. **A** Overexpression RBD outcompetes FIP2 for RAB11A binding. 293T cells, transfected with specific plasmids for 36 h, underwent co-IP assays using anti-Myc magnetic beads. Subsequent WB analysis revealed the competitive binding of RBD with FIP2 to RAB11A. **B** Overexpression RBD hinders N protein interaction with RAB11A. 293T cells, transfected with relevant plasmids for 36 h, were subjected to co-IP assays. Utilizing anti-Myc magnetic beads, the assays demonstrated RBD-mediated suppression of N protein binding to RAB11A. **C** Overexpression RBD inhibits HPIV3 infection. Hela cells, transfected with Flag-mCherry or Flag-mCherry-RBD plasmids, were infected with HPIV3 (MOI = 0.01) for 24 h. WB analysis showcased reduced viral protein levels, and TCID_50_ quantification confirmed lower viral titers in the presence of RBD. **D** and **E** Dose-dependent inhibition of HPIV3 infection by RBD and Tat peptides. HeLa cells, infected with HPIV3 (MOI = 0.01), received escalating concentrations of RBD (**D**) or Tat (**E**). At 24 hpi, WB analysis depicted the dose-dependent impact on viral protein levels, and TCID_50_ quantification confirmed the antiviral efficacy. **F** Antiviral potential of diverse peptides against HPIV3. HeLa cells, infected with HPIV3 (MOI = 0.01), were treated with escalating concentrations of various peptides. At 24 hpi, TCID_50_ quantification revealed the inhibitory effects on viral titers. **G** Enhanced designs of RBD peptides exhibit superior antiviral activity. Improved RBD peptide designs demonstrated heightened efficacy, as evidenced by TCID_50_ quantification of viral titers. **H** YT-DRI inhibits coinfection of HPIV3, RSV, and IAV in A549 cells. A549 cells were synchronously infected with a mixed inoculum of HPIV3 (MOI = 0.01), RSV (MOI = 0. 1), and IAV (MOI = 0.01), received 5 μM YT-DRI treatment. At 24 hpi, RT-qPCR analysis revealed suppressed viral RNA levels, underscoring the broad-spectrum antiviral activity. All experiments were independently replicated at least twice with consistent results. Statistical significance (*p < 0.05, **p < 0.01,  ***p < 0.001, ****p < 0.0001 ) was determined by two-sided unpaired t-test. Data are presented as means ± SEM

Having established the critical role of the RAB11A–FIP2 complex in facilitating efficient vRNP trafficking, we pursued a strategic approach to inhibit RAB11A-dependent infection by specifically targeting this interaction. To this end, we engineered a fragment of the RAB11A-binding domain (RBD) of FIP2, incorporating a Flag tag and monomeric red fluorescent protein (mCherry) to the amino terminus. We then assessed whether the RBD could effectively outcompete FIP2 for binding to RAB11A by evaluating the interaction between FIP2 and active RAB11A in 293T cells using co-IP assays. Overexpression of mCherry-RBD, compared to mCherry used as a control, resulted in a significant decrease in the levels of FIP2 co-purifying with RAB11A (Fig. 4A). To further validate whether mCherry-RBD overexpression disrupted the N-RAB11A interaction by inhibiting the formation of the RAB11A–FIP2 complex, we conducted a similar co-IP assay. The results revealed that the N-RAB11A interaction was nearly undetectable in the presence of mCherry-RBD, while it remained unchanged with mCherry alone (Fig. 4B). To evaluate the antiviral activity of mCherry-RBD overexpression, HeLa cells were transfected with plasmids expressing either mCherry or mCherry-RBD and subsequently infected with HPIV3. Notably, mCherry-RBD—unlike mCherry—exhibited a strong inhibitory effect on HPIV3 infection (Fig. 4C). These findings collectively indicate that mCherry-RBD, rather than mCherry alone, competes for FIP2 binding to RAB11A, thereby disrupting the N-RAB11A interaction and effectively inhibiting HPIV3 infection.

### Development of the FIP2-RBD-Tat peptide as an antiviral therapy against HPIV3

We subsequently explored the potential of the 28-amino acid FIP2-RBD sequence as a candidate peptide therapeutic. The FIP2-RBD peptide was C-terminally conjugated with the Tat_49–57_ peptide, a well-characterized cell-penetrating peptide (CPP) known for enhancing cellular uptake and exhibiting a favorable safety profile in clinical studies [46]. Notably, RBD-Tat (hereafter referred to simply as “RBD”) efficiently penetrated HeLa cells, irrespective of HPIV3 infection, as demonstrated in Fig. S4A. To evaluate the cytotoxicity of RBD, we employed Cell Counting Kit-8 (CCK-8) assays. The results revealed that the 50% cytotoxic concentration (CC_50_) of RBD in HeLa cells and other cell lines exceeded 75 μM, indicating minimal cytotoxic impact (Fig. S4, B to F). To assess the antiviral activity of RBD, we examined viral protein accumulation via immunoblotting and measured viral titers in the supernatant using the TCID_50_ method in HPIV3-infected HeLa cells. The findings demonstrated potent, dose-dependent antiviral activity of RBD against HPIV3, with an estimated 50% inhibitory concentration (IC_50_) of 3.25 μM (Fig. 4D). In contrast, the negative control peptide Tat exhibited no discernible antiviral effect on HPIV3 infection, even at elevated concentrations (Fig. 4E).

Further investigations into the inhibitory effects of RBD on HPIV3 were conducted across various cell lines, including A549, MK2, and MDCK cells, by measuring viral titers in the supernatant. While maintaining low cytotoxicity in these cell lines, RBD effectively inhibited HPIV3 infection in a dose-dependent manner across all tested cell types. As illustrated in Fig. S4, G to I, RBD treatment demonstrated significant anti-HPIV3 activity in all cell lines, with IC_50_ values of 3.48 μM in A549 cells, 2.71 μM in MK2 cells, and 4.88 μM in MDCK cells. These results indicate that targeting the RAB11A–FIP2 complex with RBD represents a promising antiviral strategy against HPIV3.

### YT-DRI: a potent peptide targeting the RAB11A–FIP2 complex for broad-spectrum antiviral therapy against respiratory RNA viruses

In an effort to enhance the design of RBD peptides, we synthesized a series of variants based on the RBD sequence to identify the minimal critical residues essential for binding to RAB11A. Insights were drawn from the crystal structure of the RAB11-FIP2 complex, which elucidated the structural basis for RAB11 recognition by FIP2 [35]. Despite the divergent amino acid sequences of FIPs, the C-terminal region containing the RBD is conserved. This conservation prompted us to hypothesize that a shorter FIP2-RBD could exhibit greater structural stability, thereby enhancing its ability to outcompete other FIPs for binding to RAB11A. To pursue this hypothesis, we synthesized a range of RBD truncation mutants. Among these, the peptide YT demonstrated the most potent activity, exhibiting an IC_50_ of 1.99 µM (Fig. 4F). To further optimize YT, we reversed its sequence and replaced the amino acids with D-isomers, which are known for their enhanced stability and superior antiviral efficacy [47–49]. The resulting peptide, designated YT-DRI, not only displayed reduced cytotoxicity (Fig. S5, A to D) but also demonstrated excellent anti-HPIV3 activity in a dose-dependent manner, with IC_50_ values of 0.94 µM in HeLa cells (Fig. 4G), 0.57 µM in A549 cells, and 1.46 µM in MDCK cells (Fig. S5, E and F). Given the conservation of the RAB11-dependent recycling pathway among many negative-sense RNA viruses, including RSV and IAV [37, 39, 41–43, 50], it is reasonable to speculate that YT-DRI could exhibit broad-spectrum antiviral efficacy. To test this, we treated RSV-infected (Fig. S5, G to J) and IAV-infected cells (Fig. S5, K to M) with YT-DRI, revealing significant antiviral activity against both viruses. The CC_50_ for YT-DRI was determined to be greater than 150 µM in the tested cells (Fig. S5, A to D), yielding a favorable selectivity index (SI = CC_50_/IC_50_) greater than 200 for HPIV3, RSV, and IAV (Table 1), reflecting its robust therapeutic potential and favorable safety profile. Considering the frequent occurrence of viral co-infection, we deemed it essential to evaluate the antiviral activity of YT-DRI in the context of simultaneous HPIV3, RSV, and IAV infections. To achieve this, we infected human lung-derived A549 cells with a combination of HPIV3, RSV, and IAV inoculum. Using quantitative reverse transcription-PCR (RT-qPCR) to measure viral RNA accumulation, we found that YT-DRI, compared to the negative control peptide Tat at equivalent concentrations, markedly impeded the replication of HPIV3, RSV, and IAV in vitro (Fig. 4H). Similar results were obtained in HeLa and MDCK cells (Fig. S5, N and O). Collectively, these findings underscore the potential of targeting the RAB11A–FIP2 complex as a promising strategy for developing broad-spectrum antiviral therapies against diverse respiratory RNA viruses. Peptides such as YT-DRI demonstrate considerable clinical promise for further development.

**Table 1:** Anti-respiratory RNA viruses peptides

Virus	HPIV3								
Cell type	HeLa		A549		MK2		MDCK		
Peptide	IC_50_	SI	IC_50_	SI	IC_50_	SI	IC_50_	SI	CC_50_
RBD	3.25	> 23.08	3.48	> 21.55	2.71	> 27.68	4.88	> 15.37	> 75
YT-DRI	0.94	> 159.57	0.57	> 263.16	ND	ND	1.46	> 102.74	> 150

Virus	RSV								
Cell type	HeLa		A549		HEp-2		MDCK		
Peptide	IC_50_	SI	IC_50_	SI	IC_50_	SI	IC_50_	SI	CC_50_
YT-DRI	0.68	> 220.59	0.37	> 405.41	0.22	> 681.82	0.46	> 326.09	> 150

Virus	IAV						
Cell type	HeLa		A549		MDCK		
Peptide	**IC**_**50**_	SI	IC_50_	SI	IC_50_	SI	CC_50_
YT-DRI	0.78	> 192.31	0.6	> 250	0.42	> 357.14	> 150

*ND* not determined, *IC*_*50*_ CC_50_(μM), *SI* CC_50_/IC_50_

### YT-DRI treatment inhibits virus replication through interrupting the RAB11A-associated pathway

**Fig. 5:**
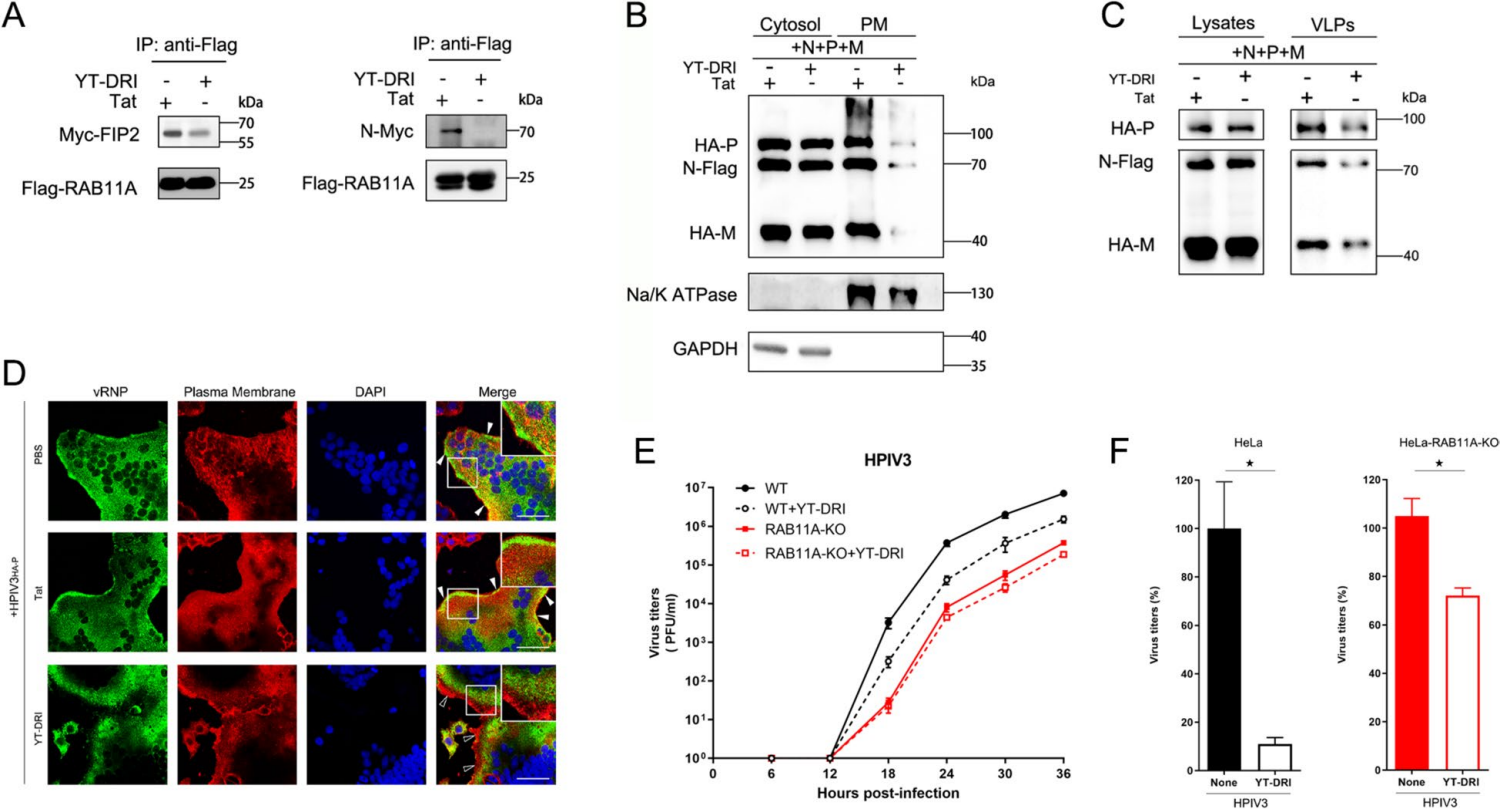
YT-DRI inhibits HPIV3 replication through disrupting the RAB11A–FIP2 complex. **A** YT-DRI disrupts N-RAB11A interaction by competing with FIP2 for RAB11A binding. 293 T cells were individually transfected with plasmids encoding FIP2, the N protein, and RAB11A for 36 h, after which their lysates were mixed for in vitro co-IP analysis. The co-IP assays, using anti-Flag magnetic beads, showed that YT-DRI interferes with N-RAB11A binding, effectively competing with FIP2. **B** and **C** YT-DRI inhibits vRNP distribution and VLPs production. 293 T cells, transfected with relevant plasmids for 48 h, underwent cytosol and PM fractionation, with supernatants analyzed for VLPs. WB analysis, using GAPDH and Na/K ATPase as markers, revealed YT-DRI's impact on vRNP distribution (**B**) and VLPs production (**C**). **D** YT-DRI impedes HPIV3 infection by disrupting vRNP distribution. HeLa cells, infected with HPIV3_HA-P_ (MOI = 0.01), received 5 μM YT-DRI or Tat treatment for 24 h. Immunofluorescent detection of vRNP displayed altered distribution patterns, with YT-DRI hindering vRNP clusters at the PM. **E** The growth curve of the HPIV3 with or without treatment of YT-DRI on HeLa (WT and RAB11A-KO) cells. The HeLa cells were infected with HPIV3 (MOI = 0.05) and treated YT-DRI at a final concentration of 5 μM (∼5IC_50_). The supernatants were assayed for viral titers at 6 h, 12 h, 18 h, 24 h, 30 h, and 36 h post-infection by TCID_50_. **F** Statistical analysis of YT-DRI's antiviral impact at 24 h post-infection. The statistical significance of YT-DRI's antiviral effect in RAB11A-depleted cells was determined by two-sided unpaired t-test. All experiments were independently replicated at least twice with consistent results. Statistical significance (*p < 0.05) was determined by two-sided unpaired t-test. Data are presented as means ± SEM

YT-DRI peptide has the potential to disrupt the N-RAB11A interaction by competing with FIP2 for binding to RAB11A. To investigate this, we individually expressed FIP2, the N protein, and RAB11A in 293T cells, then mixed their lysates for in vitro co-IP analysis. The results indicated that the interaction between FIP2 and RAB11A was significantly attenuated in the presence of YT-DRI, in contrast to the control peptide Tat (Fig. 5A). Notably, the N-RAB11A interaction became undetectable, highlighting YT-DRI's capacity to suppress viral infection in vitro. We further examined whether YT-DRI could decrease vRNP levels in the PM fraction. Our data revealed reduced levels of the N, P, and M proteins in both the PM fraction (Fig. 5B) and VLPs (Fig. 5C) following YT-DRI treatment compared to the Tat-treated control group. To confirm YT-DRI's targeting of vRNP trafficking as the mechanism for its antiviral effect, we investigated its impact on HPIV3_HA-P_ infection, with vRNP localization assessed through immunostaining. YT-DRI treatment, as opposed to the negative control peptide Tat, resulted in the formation of large aggregates of vRNP in the cytoplasm, significantly impeding its trafficking toward the PM (Fig. 5D). These findings demonstrate that YT-DRI competes with FIP2 for RAB11A binding, thereby disrupting the N-RAB11A interaction and inhibiting vRNP trafficking.

To ascertain whether YT-DRI effectively targets the RAB11A-associated pathway to exert its antiviral effects, we analyzed the impact of YT-DRI treatment on HPIV3 infection in both wild-type (WT) and RAB11A-KO HeLa cells. Given the absence of RAB11A in RAB11A-KO cells, we anticipated that these cells would be less responsive, or unresponsive, to YT-DRI treatment. Our results demonstrated that YT-DRI addition to the culture medium resulted in a significant reduction in viral growth in WT cells, while the effect on HPIV3 in RAB11A-KO cells was moderate and statistically insignificant (Fig. 5E). Thus, these results provide compelling evidence that the antiviral activity of YT-DRI is primarily dependent on RAB11A. The statistical analysis for 24 hpi is presented in Fig. 5F. Additionally, we found that YT-DRI had no significant antiviral effect on RSV or IAV infections in RAB11A-KO HeLa cells (Fig. S6, A and B), further supporting that its antiviral activity is RAB11A-dependent. Notably, the replication of Enterovirus 71 (EV71) in both RAB11A-KO and WT HeLa cells was comparable (Fig. S6C), indicating that RAB11A is not required for EV71 replication. Furthermore, we observed no significant differences in EV71 replication in YT-DRI-treated cells compared to untreated cells (Fig. S6D), confirming that YT-DRI does not exert off-target effects. Collectively, these results demonstrate that YT-DRI treatment inhibits viral replication by specifically targeting the RAB11A–FIP2 complex and disrupting the RAB11A-associated pathway.

### YT-DRI: a broad-spectrum antiviral peptide for inhibiting respiratory RNA viruses in vivo

Considering the potent antiviral effects of YT-DRI observed in vitro, we further investigated its efficacy against respiratory RNA viruses using mouse models. To explore the preclinical potential of YT-DRI, we evaluated its in vivo distribution and therapeutic effects. Intraperitoneal (i.p.) injection of fluorescently labeled Cy5-YT-DRI in BALB/c mice demonstrated widespread distribution throughout the body, with notable accumulation in organs such as the lungs, heart, liver, kidneys, and brain (Fig. S7, A to G). The i.p. administration of YT-DRI effectively targeted the lower respiratory tract and facilitated transport across the blood–brain barrier, suggesting its potential as a treatment for both systemic and cerebral infections.

**Fig. 6:**
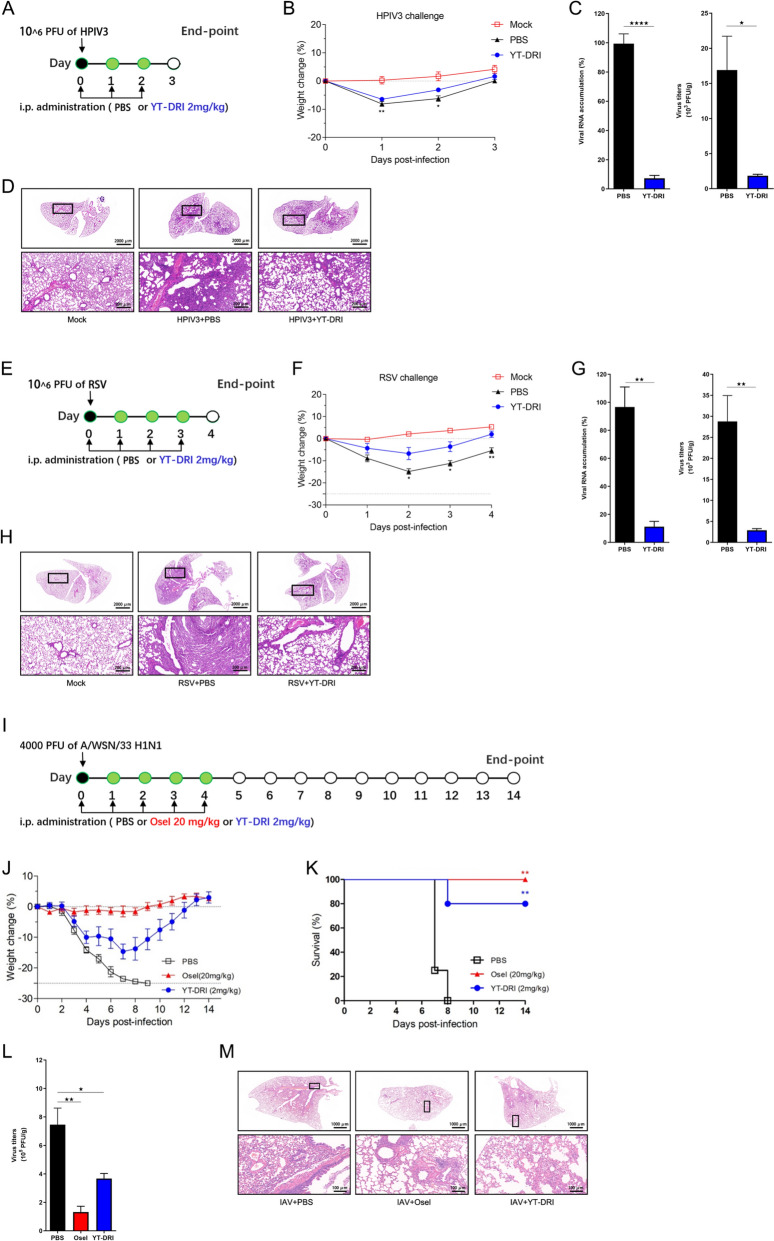
In Vivo evaluation of YT-DRI against respiratory viral infections. **A** Experimental design for HPIV3 infection. 6–8-week nude BALB/c mice were intranasally infected with 2 × 10^6^ PFU of HPIV3 per mouse and treated with PBS or YT-DRI (2 mg/kg) via intraperitoneal injection, administered once per day from Day 0 to Day 2. Mouse body weight was monitored daily for 4 days. **B** Body weight changes in HPIV3 infection. Graphical representation illustrating the body weight changes of mice in different groups over the course of the experiment. **C** and **D** Viral RNA copies, the infectious titers and the lung histopathology. At Day 3, animals were euthanized, lung tissues were collected, homogenized. Viral RNA level was measured with quantitative RT-qPCR and virion load was titered by TCID_50_ assay (**C**). Histopathology of lung tissues was assessed through hematoxylin and eosin (H&E) staining (**D**). **E** Experimental design for RSV infection. 6–8-week nude BALB/c mice were intranasally challenged with 3 × 10^6^ PFU of RSV and treated with PBS or YT-DRI (2 mg/kg) via intraperitoneal injection. Treatment occurred once per day from Day 0 to Day 3. Daily monitoring of body weight was conducted for 5 days. **F** Body weight changes in RSV infection. Graphical representation illustrating the body weight changes of mice in different groups over the course of the experiment. **G** and **H** Viral RNA copies, the infectious titers and the lung histopathology in RSV infection. At Day 4, animals were euthanized, lung tissues were collected, homogenized, and supernatants were tested by quantitative RT-qPCR and TCID_50_ (**G**). Histopathology of lung tissues was assessed through H&E staining (**H**). **I** Experimental design for WSN virus infection. 6–8-week BALB/c mice were intranasally challenged with 4,000 PFU of WSN virus and treated with PBS, Oseltamivir (20 mg/kg), or YT-DRI (2 mg/kg) via intraperitoneal injection. Treatment occurred once per day from Day 0 to Day 4. Daily monitoring of body weight and survival was conducted for 14 days or until body weights lost more than 25%. **J** Body Weight Changes in WSN Virus Infection. Graphical representation illustrating the body weight changes of mice in different groups over the course of the experiment. **K** Mouse survival was observed and recorded daily until 14 dpi. **L** and **M** In addition, another three groups of mice were administrated with the same methods except that these mice were sacrificed at Day 4 to determine the virus load via RT-qPCR (**L**) and the lung histopathology (**M**). All experiments were independently repeated at least twice with reproducible results. Statistical significance (*p < 0.05, **p < 0.01, ***p < 0.001, ****p < 0.0001 ) was determined by two-sided unpaired t-test for statistical significance and log-rank test for survival curves. Data are presented as means ± SEM

Six to eight-week-old nude BALB/c mice were intranasally infected with 2 × 10^6^ PFU of HPIV3 per mouse on day 0 and subsequently treated with YT-DRI (2 mg/kg) or phosphate-buffered saline (PBS) as a control, with treatments administered once daily for a subsequent period of 2 days (Fig. 6A). Mice treated with YT-DRI experienced less weight loss compared to those receiving PBS (Fig. 6B). Mice were euthanized at day 3 post-infection (dpi), and lungs were harvested. Viral RNA levels in the lung tissue were measured using quantitative RT-qPCR, and virion loads were assessed through TCID_50_ assays (Fig. 6C). Treatment with YT-DRI (2 mg/kg) significantly decreased both viral RNA copies and infectious titers in the lung (Fig. 6C). Histological analysis revealed reduced lung inflammation in YT-DRI-treated mice compared to PBS-treated controls, further supporting its efficacy in mitigating lung injury following HPIV3 infection (Fig. 6D). These findings collectively demonstrate that YT-DRI has robust in vivo therapeutic efficacy against HPIV3 infection.

To assess the effectiveness of YT-DRI against respiratory syncytial virus (RSV), nude BALB/c mice were intranasally infected and subsequently treated with either YT-DRI (2 mg/kg) or PBS, with treatments administered once daily for a subsequent period of 3 days (Fig. 6E). At the peak of viral infection on day 4 post-infection (dpi), YT-DRI treatment resulted in reduced body weight loss (Fig. 6F), lower viral RNA copies, and decreased infectious titers (Fig. 6G), along with diminished lung tissue damage (Fig. 6H), demonstrating its therapeutic potential against RSV infection.

In a lethal model of influenza A virus (IAV, WSN strain) infection, low-dose YT-DRI (2 mg/kg) administered i.p. yielded antiviral effects comparable to those of 20 mg/kg of Oseltamivir (Osel), resulting in reduced body weight loss and a survival rate of 80% (Fig. 6J and K). Osel served as a positive control, providing complete protection against morbidity and mortality. Additionally, three other groups of mice were treated similarly but were sacrificed at 4 dpi to assess viral load in the lungs via RT-qPCR and histological analysis. Similar to Osel treatment, YT-DRI decreased viral RNA copies and resulted in milder lung inflammation compared to PBS-treated controls (Fig. 6L and M).

Furthermore, acute toxicity tests revealed that YT-DRI does not cause significant harm to the health of mice. Even at doses five times higher than those used for therapeutic protection, no significant changes were observed in body weight or histopathological morphology (Fig. S8). Collectively, these results highlight the broad-spectrum antiviral efficacy of YT-DRI against HPIV3, RSV, and IAV in vivo, supporting its potential as an effective therapeutic agent targeting the RAB11A-associated pathway.

## Discussion

In this study, we elucidate the critical roles of actin microfilaments and RAB11A in the late stages of HPIV3 infection. We demonstrate the inhibitory efficacy of peptides, particularly YT-DRI, that target the RAB11A–FIP2 complex against HPIV3, RSV, and IAV (Table 1). Furthermore, we present multiple lines of evidence supporting the RAB11A-dependent antiviral effect. Notably, replication of EV71 in both wild-type and RAB11A-knockout HeLa cells was similar (Fig. S6C), indicating that RAB11A is not necessary for EV71 replication. Consistently, there were no significant differences in EV71 replication between YT-DRI-treated and untreated cells (Fig. S6D), suggesting that YT-DRI does not exert off-target effects. Collectively, these results demonstrate that YT-DRI treatment inhibits viral replication by targeting the RAB11A–FIP2 complex and disrupting the RAB11A-associated pathway. These findings highlight the potential of targeting the RAB11A-dependent recycling pathway as a promising strategy for developing broad-spectrum antiviral drugs effective against various respiratory RNA viruses. Overall, YT-DRI, as a host-targeting antiviral, holds promise in avoiding drug resistance caused by viral mutations, offering a potential advantage over conventional pharmaceuticals. Moreover, RAB11A-targeting drugs can take various forms, such as peptides, nucleic acids, nucleoside analogs, or small-molecule compounds, broadening the scope of potential antiviral agents.

To further validate the specificity of our RAB11A-targeting peptide, we selected EV71, a positive-sense RNA virus, as a control. Our results demonstrated that EV71 replication was unaffected by RAB11A knockout (Fig. S6C), and the peptide showed no inhibitory effect on EV71 in wild-type cells (Fig. S6D). These results confirm that the antiviral activity of our peptide is specifically dependent on the RAB11A signaling pathway, rather than non-specific cytotoxicity.

Our findings also raise important questions about the conservation of the RAB11A–FIP2 pathway among other respiratory viruses. For example, while our study focused on HPIV3, RSV, and IAV, influenza B virus (IBV) is another significant human respiratory pathogen that may rely on RAB11A for replication. Future studies should explore the efficacy of our peptide against IBV to further establish its broad-spectrum potential.

From the results of co-localization studies (Fig. S2), RAB11A in its constitutively active form (RAB11A_CA_) has a unique interaction with vRNP among other constitutively active RAB proteins, suggesting that RAB11A plays a crucial role in vRNP trafficking within the RAB GTPase family. Our results align with prior evidence highlighting the dependence of HPIV3 on RAB11A-associated endosomal trafficking, a phenomenon observed in various viral infections [37, 38, 43, 50]. However, the mechanism of vRNP-RAB11A interaction is unique to HPIV3. To decipher the mechanism, we employed co-IP assays and found that the interaction between vRNP and RAB11A was possibly mediated by the N protein (Fig. S3A). Upon further dissecting this interaction, we found that the N protein interacts only with the active form of RAB11A, particularly with RAB11A_CA_ (Fig. 3A), consistent with the microscopy data shown in Fig. 3B. Interestingly, RAB11A co-localized with vRNP at the PM; however, RAB11A_CA_ induced vRNP aggregate formation, which hindered its migration to the PM. Testing vRNP levels in the PM fraction and supernatant (Fig. 3C) revealed a reduced vRNP level in the presence of RAB11A_CA_, suggesting that RAB11A_CA_ overexpression impairs the recycling capacity of infected cells, potentially limiting vRNP accumulation at the PM for virion formation. To validate our hypothesis, we examined the effect of RAB11A_CA_ on HPIV3 replication (Fig. 3D). Our findings revealed a significant, dose-dependent reduction in supernatant titers of cells expressing RAB11A_CA_, indicating that an optimal interaction between the N protein and RAB11A is essential for viral replication. Given the importance and complexity of the vRNP-RAB11A interaction, we questioned whether FIP2, a multifunctional RAB11-family interacting protein, also played a role in the intricate relationship between active RAB11A and vRNP, beyond its role in bridging recycling endosomes and actin filaments. During IAV infection, vRNPs outcompeted FIPs for binding to RAB11A, resulting in an altered distribution of RAB11A [39, 42, 45]. To determine FIP2's role in the interaction between HPIV3 vRNP and RAB11A, we examined the binding of the N protein to RAB11A using co-IP (Fig. 3H and I). In contrast with the competitive relationship between IAV RNPs and FIPs, our results revealed a positive role for FIP2 in the N-RAB11A interaction, providing novel insights into the interplay among vRNP, FIPs, and RAB11A. To further confirm the importance of the RAB11A–FIP2 interaction in vRNP transport and HPIV3 infection, we analyzed VLP production and replication in FIP2-KO HeLa cells in the presence of FIP2 or a FIP2 variant lacking the RBD domain (FIP2_△RBD_) (Fig. 3J and K). The results showed that only FIP2, not the RBD-deleted variant FIP2_△RBD_, could restore both VLP production and replication. Together, these findings indicate that the RAB11A–FIP2 complex is essential for RAB11A-dependent vRNP trafficking.

HPIV3 replicates effectively in the respiratory tracts of cotton rats, ferrets, lambs, and African green monkeys [6, 51–54]. However, these species are less suitable as preclinical models compared to mice due to their larger size, higher maintenance costs, limited accessibility, and the complexity involved in their management and care. BALB/c mice have been utilized in various studies to evaluate immune responses to protein subunit vaccines against HPIV3 [51, 52], but no robust challenge models for HPIV3 have been established in immunocompetent mice, implying that they may be insufficiently susceptible to HPIV3 infections. The BALB/c mouse model, which is currently utilized for investigating RSV immunopathology, exhibits semi-permissive characteristics towards the virus [55–57]. Respiratory viruses, such as RSV and HPIV3, can lead to severe pulmonary disease and pneumonia in immunocompromised individuals [58]. This increased susceptibility to viral infections during the post-transplant period is attributed to the impaired T cell immunity, which has not yet been fully restored [59–63]. Studies comparing RSV infection and inflammatory responses in immunocompetent BALB/c mice versus immune-deficient nude mice have revealed that nude mice exhibit elevated viral replication and increased airway inflammation following infection [64]. Nude mice are inbred mice containing the same genetic background as the BALB/c mice, except for their cellular immune-deficiencies, suggesting that nude mice may be a good immunocompromised model for studying severe HPIV3 and RSV infection [65]. Consequently, we have chosen nude BALB/c mice as our model for HPIV3 (Fig. 6A to D) and RSV (Fig. 6E to H) infections to assess the in vivo therapeutic efficacy of YT-DRI.

One limitation of this study is that our current understanding is largely derived from studies involving two of these viruses. Co-infection with IAV and HPIV2 can boost IAV replication by aiding the distribution of its genome to the nuclei of merged cells [66]. With IAV and RSV co-infection, it results in the creation of hybrid viral particles that contain both viral genomes and display a combination of their glycoproteins on their surface, thereby expanding the receptor tropism of these particles [67]. However, the dynamics involving all three viruses may be much more complicated. Furthermore, despite the possibility of simultaneous or sequential viral co-infections, we opted to infect cells simultaneously with a mixture of three viruses—HPIV3, RSV, and IAV—to simplify our experimental approach and ensure consistent, reproducible outcomes. Another limitation of our study is the absence of suitable animal models capable of investigating co-infections involving multiple respiratory RNA viruses. This limitation restricts our ability to assess the in vivo antiviral efficacy of YT-DRI treatment in scenarios where multiple pathogens simultaneously infect the same host.

Additionally, our focus on respiratory RNA viruses may not fully encompass the broader implications of RAB11A-dependent cellular trafficking pathways, which are implicated in the infection cycles of various viruses. Notably, we have demonstrated that YT-DRI can traverse the blood–brain barrier to enter murine brains, suggesting that systemic and cerebral infections induced by RAB11A-dependent viruses may also be amenable to treatment with YT-DRI. Another concern regarding our findings is that, while antiviral peptides show considerable promise, ongoing peptide engineering is essential for optimizing their efficiency, selectivity, and pharmacological properties. Furthermore, the integration of antiviral peptides with other drug classes in a comprehensive treatment regimen warrants further investigation.

## Conclusion

Respiratory viruses such as respiratory syncytial virus (RSV), influenza A virus (IAV), and human parainfluenza virus type 3 (HPIV3) are major causes of acute respiratory infections, with co-infections posing a significant public health challenge. Current vaccines and antiviral drugs are virus-specific, highlighting the urgent need for broad-spectrum therapeutics. In this study, developed a series of peptides targeting the RAB11 pathway, a critical component of host vesicular trafficking essential for viral replication. Among these, the optimized peptide YT-DRI demonstrated potent antiviral activity against HPIV3, RSV, and IAV in vitro and in vivo, and effectively suppressed co-infections in cellular models. Importantly, the antiviral efficacy of YT-DRI was abolished in RAB11A-knockout cells, confirming its specificity for the RAB11-associated pathway. Our findings suggest that YT-DRI inhibits viral replication by disrupting RAB11A-dependent trafficking, offering a promising strategy for developing broad-spectrum antivirals against respiratory infections. Future studies should explore its efficacy against other RAB11-dependent viruses, such as influenza B virus (IBV) and coronaviruses, to further establish its therapeutic potential and advance our understanding of RNA biology in viral infections.

## Materials and methods

### Cell culture

Human cervical cancer cell line HeLa, Human Embryonic Kidney cells HEK293T, Human lung adenocarcinoma cell line A549, Rhesus Monkey Kidney Epithelial cells LLC-MK2, Madin-Darby Canine Kidney cells MDCK, Human Epidermoid carcinoma cells HEp-2, and Rhabdomyosarcoma cells RD were procured from the China Center for Type Culture Collection (CCTCC) and cultured in Dulbecco’s modified Eagle’s medium (DMEM, Gibco), supplemented with 10% fetal bovine serum (FBS, WISENT) and 1% penicillin–streptomycin (Gibco) at 37 °C with 5% CO_2_. The knock-out cell lines were generated using the CRISPR/Cas9 gene-editing system. Synthesized sgRNAs were cloned into the lentiCRISPRv2 vector. 293T cells were co-transfected with lentiCRISPRv2-target sgRNA, pMD2.G, and psPAX2 for 48 h. Lentivirus supernatants were harvested and filtered through a 0.45-mm Millipore filter. HeLa or 293T cells were transduced with lentiviruses expressing the sgRNAs for 48 h at 37 °C and then selected with 2 mg/mL puromycin for 3–6 days. Subsequently, cells were seeded in 96-well plates for single-cell sorting. Single-cell clones were screened and validated by Western blotting to assess protein expression. The primers of sgRNAs were listed in Table S1.

### Viruses

HPIV3, recombinant HPIV3_HA-P_, and HPIV3 clinical strain VR93 were propagated in MK2 cells through inoculation at a multiplicity of infection (MOI) of 0.1. RSV A2 strain was propagated in HEp-2 cells by inoculation at an MOI of 0.1. The A/WSN/33 virus was propagated in MDCK cells. EV71 was amplified by infecting RD cells.

### Mice

All animals were procured from the Beijing Vital River Laboratory Animal Technology Co (Beijing, China) and housed in the specific pathogen-free animal facility. The experimental procedures were ethically reviewed and approved by the Animal Committee of Wuhan University.

### Peptides

Peptides Tat _49–57_ (RKKRRQRRR),

RBD (THIRELEDYIDNLLVRVMEETPSILRVPRKKRRQRRR),

FITC-RBD,

LP(LEDYIDNLLVRVMEETPSILRVPRKKRRQRRR), LR(LEDYIDNLLVRVMEETPSILRRKKRRQRRR), YP(YIDNLLVRVMEETPSILRVPRKKRRQRRR),

YR (YIDNLLVRVMEETPSILRRKKRRQRRR),

LT(LEDYIDNLLVRVMEETRKKRRQRRR),

YT(YIDNLLVRVMEETRKKRRQRRR),

LM(LEDYIDNLLVRVMRKKRRQRRR),

YM(YIDNLLVRVMRKKRRQRRR) were synthesized by Sangong Biotech (Shanghai).

YT-DRI (rrrrrkkrtmvrvndy, with D-amino acids in a retro reversed sequence) and Cy5-YT-DRI were meticulously synthesized by KareBay Biochem. The peptides were accurately dissolved in PBS for subsequent experiments.

### Assessment of peptide cytotoxicity

To evaluate the cytotoxicity of peptides across various cell lines, a CCK-8 assay was employed. Cells cultured overnight in 96-well plates at 37 °C were treated with increasing concentrations of peptides for 24 h. Afterward, the cell supernatant was replaced with fresh DMEM, and the addition of CCK-8 reagent to each well allowed for absorbance measurements at 450 nm after a 1-h incubation.

### Cell infection and transfection procedures

For infection experiments, cells were exposed to viruses for a duration of 2 h, after which the medium was substituted with fresh DMEM containing 4% FBS, along with the addition of drugs or peptides. Harvesting of samples for various assays occurred when infected cells exhibited noticeable cytopathic effects (CPE). In the case of transfection, cells underwent plasmid transfection using Lipofectamine 2000 (Invitrogen) following the manufacturer’s guidelines. Subsequent treatments were applied as necessary.

### Virus titration

To quantify the number of infectious virions in culture supernatants, the median tissue culture infectious dose (TCID_50_) assay was employed. This assay involved the addition of virus samples at varying dilutions to monolayer cells in 96-well plates. The count of wells displaying cytopathic effect (CPE) in each dilution was recorded to ascertain the dilution point at which 50% of the wells exhibited CPE. Viral titers were subsequently calculated using the Reed-Muench method.

### RNA extraction and RT-qPCR analysis

Total RNA extraction was conducted employing TRIzol reagent (Invitrogen) as per the manufacturer’s guidelines. Subsequently, the extracted RNA was reverse transcribed using a cDNA First Strand Synthesis kit (ABclonal). The quantitative reverse transcription-polymerase chain reaction (RT-qPCR) was executed using 2 × universal SYBR green fast qPCR mix (ABclonal). The primers were listed in Table S1. RT-qPCR analysis was performed on a Bio-Rad CFX96 Touch real-time PCR detection system.

### HPIV3 binding and internalization assay

For the binding assay, HPIV3 was added to pre cooled cells for 1 h at 4 °C. After incubation, HPIV3 was removed, and the cells were washed 3 times with phosphate-buffered saline (PBS) to remove unbound virions. The cells were harvested and then the total RNA was extracted for RT-qPCR analysis. The relative cell binding of HPIV3 was calculated relative to GAPDH and normalized to control samples (wild-type cells). For the internalization assay, cells were first treated as for a binding assay and then incubated at 37 °C with for 1 h to allow internalization. After incubation, the supernanant was removed, and the cells were washed 3 times with PBS to remove virions that had not been internalized. The cells were harvested and then the total RNA was extracted for RT-qPCR analysis.

### SDS-PAGE and western blot

Cells were harvested and lysed in ice-cold lysis buffer for 30 min, followed by centrifugation at 4 °C at 12,000×*g* for 30 min. The supernatants obtained were collected and boiled with SDS-PAGE loading buffer at 100 °C for 10 min. Prepared samples were resolved on 10% SDS-PAGE, and proteins were subsequently transferred onto nitrocellulose membranes. Prior to antibody incubation, membranes were blocked with 5% skim milk in PBS with 0.1% Tween 20 (PBST) for 30 min. Primary antibodies were then applied, followed by incubation with HRP-conjugated goat anti-mouse or -rabbit IgG secondary antibodies. The primary antibodies included mouse anti-Flag (1:10,000, MBL), mouse anti-Myc (1:10,000, MBL), mouse anti-HA (1:10,000, MBL), rabbit anti-Myc (1:5,000, MBL), mouse anti-HN (1:2500, Abcam), rabbit anti-RAB11A (1:1000, Proteintech), rabbit anti-RAB11A–FIP2 (1:1000, Proteintech), rabbit anti-Na/K ATPase (1:1000, ABclonal), mouse anti-GAPDH (1:1000, ABclonal), and mouse anti-β-tubulin (1:1000, ABclonal). Secondary antibodies, goat anti-mouse IgG and goat anti-rabbit IgG, were used at a 1:10,000 dilution. Membranes were visualized using the Fujifilm LAS-4000 imaging system.

### Co-immunoprecipitation (co-IP) assay

293T cells were transfected with the indicated plasmids for 36 h, and cell lysates were prepared using lysis buffer as described in the Western blot (WB) analysis section. The supernatants were collected and incubated with anti-Flag or anti-Myc magnetic beads (MCE) following the manufacturer’s instructions for co-IP. The immunoprecipitated proteins were then analyzed via WB.

### Virus-like particle budding assays

293T cells were transfected with the indicated plasmids for 48 h. The cell lysates were prepared as described for the Western blot (WB) analysis. The supernatant was harvested and clarified by centrifugation at 12,000×*g* for 5 min. Subsequently, the supernatant was pelleted through a 20% sucrose cushion at 4 °C at 35,000 rpm for 2 h. The VLP pellets obtained were resuspended in PBS and boiled with SDS-PAGE loading buffer at 100 °C for 10 min. Both the cell lysates and the supernatants were then subjected to WB analysis.

### Membrane association assay

293T cells, cultured in 6-cm dishes, were transfected with the indicated plasmids for 24 h. Cell collection was performed through centrifugation at 500×*g* for 5 min at 4 °C. Subsequently, cytosol, organelle membrane, and plasma membrane-associated proteins were extracted sequentially using the MinuteTM plasma membrane protein isolation and cell fractionation kit (INVENT), following the manufacturer's instructions. The cytosol and plasma membrane proteins obtained were boiled with SDS-PAGE loading buffer at 100 °C for 10 min and subjected to Western blot (WB) analysis. β-tubulin and sodium–potassium ATPase were utilized as markers for cytosol and plasma membrane proteins, respectively.

### Immunofluorescence staining and confocal microscopy

HeLa cells were cultured on coverslips in 24-well plates overnight and subjected to transfection or/and virus infection as indicated. Following this, cells were fixed with 4% paraformaldehyde for 20 min at room temperature (RT) and permeabilized with 0.2% Triton X-100 for 20 min at RT. Subsequently, cells were blocked with 3% bovine serum albumin (BSA) in PBS for 30 min at RT. The cells were then incubated with primary antibodies diluted in 1% BSA, followed by incubation with secondary antibodies, also diluted in 1% BSA. Nuclei were counterstained with DAPI, and plasma membrane were stained with Dil. Confocal microscopy images were acquired using a Leica confocal microscope.

### In vivo immunofluorescence assay

Female BALB/c mice, aged 6–8 weeks, were randomly divided into two groups. One group received an intraperitoneal injection of PBS (n = 3) for background fluorescence measurement, while the other group received 5 mg/kg Cy5-YT-DRI (n = 3). Bioluminescence readings were performed at various time points using the IVIS Lumina K Series III. Following imaging, mice were euthanized, and the indicated tissues were collected for further evaluation of fluorescence values. The average radiance is expressed as the sum of photons per second from each pixel inside the region of interest per the number of pixels (p s^−1^ cm^−2^ sr^−1^).

### In vivo drug toxicity study

The 6–8 weeks old male ICR male mice, were randomly assigned into three groups and each group had 5 mice. The mice were i.p. injected with 5 mg/kg YT-DRI, 10 mg/kg YT-DRI, or the same volume of PBS for consecutive 3 days. During the experiment, body weight was recorded for 15 days. At the end of the experiment, the mice were perfused, and tissue samples of heart, liver, lung, and kidney were collected for histopathological examination.

### In vivo HPIV3 infection model and YT-DRI treatment

6–8-week female nude BALB/c mice were anesthetized with isoflurane and intranasally challenged with 2 × 10^6^ PFU of the HPIV3 clinical strain VR93 suspended in 50 μl of PBS. Subsequently, PBS or YT-DRI (2 mg/kg) was administered via intraperitoneal injection, with the drugs administered once daily for three consecutive days. Daily monitoring of body weight and clinical symptoms was conducted for 4 days (n = 5 per group). At 3 days post-infection (dpi), mice were sacrificed, and lung tissues were subjected to histopathological examination. Additionally, virus loads in lung tissues were determined via TCID_50_, and the total RNA was extracted for RT-qPCR analysis. The relative viral RNA was calculated relative to GAPDH.

### In vivo RSV infection model and YT-DRI treatment

Female nude BALB/c mice (6–8 weeks old) were anesthetized with isoflurane and intranasally challenged with 3 × 10^6^ PFU of RSV suspended in 50 μl of PBS. Subsequently, PBS or YT-DRI (2 mg/kg) was administered via intraperitoneal injection for the first time. The drugs were injected once daily for four consecutive days. Daily monitoring of body weight and clinical symptoms was conducted for 5 days (n = 5 per group). Mice were sacrificed at 4 dpi, and lung tissues were subjected to histopathological examination. Additionally, virus loads in lung tissues were determined via TCID_50_, the total RNA was extracted for RT-qPCR analysis.

### In vivo influenza A virus infection model and YT-DRI treatment

Female BALB/c mice (6–8 weeks old) were anesthetized with isoflurane and intranasally challenged with 4,000 PFU of WSN virus suspended in 50 μl of PBS, and then PBS, Oseltamivir (20 mg/kg), or YT-DRI (2 mg/kg) was administered via intraperitoneal injection for the first time. The drugs were injected once daily for five consecutive days. Daily monitoring of body weight, clinical symptoms, and mortality was conducted for 14 days or until body weight lost more than 25% (PBS group n = 4, the other groups n = 5). In addition, another three groups of mice were administrated with the same methods except that these mice were sacrificed at 4 dpi to determine the virus load in their organs via TCID_50_, and lung tissues were subjected to histopathological examination.

### Quantification and statistical analysis

GraphPad Prism 8 was employed for all statistical analyses. Each experiment was independently repeated a minimum of two times, yielding reproducible results. Students’ two-sided unpaired t-test was utilized to determine statistical significance. Survival curve analyses were conducted using the log-rank test. A p value greater than 0.05 was considered not significant (n.s.), while p values less than 0.05 were deemed statistically significant, denoted as follows: ns, p > 0.05; *, p < 0.05; **, p < 0.01; ***, p < 0.001; ****, p <0.0001 .

## Supplementary Information

Supplementary Material 1. Fig. S1. The specific effects and cytotoxicity of Nocodazole and Cytochalasin D. (**A**) Hela cells were transfected with mCherry-Lifeact-expressing plasmids. After 24 h, cells were treated with indicated concentrations of Nocodazole or Cytochalasin D for 6 h. The resulting images, captured through fluorescent microscopy, depict F-actin labeled with mCherry-Lifeact (red), microtubules stained using a mouse anti-α-tubulin primary antibody (green), and nuclei counterstained with DAPI (blue). Scale bar, 50 μm. Cell viability was assessed in HeLa (**B**) and 293T (**C**) cells treated with indicated concentrations of Nocodazole or Cytochalasin D for 6 h using the CCK-8 assay. All experiments were independently repeated at least twice with reproducible results. Statistical significance (ns, p > 0.05) was determined by two-sided unpaired t-test for statistical significance and log-rank test for survival curves. Data are presented as means ± SEM.

Supplementary Material 2. Fig. S2. The specific co-localization of RAB11A_CA_ and vRNP. HeLa cells were transfected with plasmids expressing the specified constitutively active RAB GTPases tagged with Flag, and then subsequently infected with HPIV3_HA-P_ (MOI=0.01) for 24 h. Immunofluorescent staining was conducted using rabbit anti-HA antibody (in red) and mouse anti-Flag antibody (in green), while nuclei were counterstained with DAPI. Scale bar, 50 μm.

Supplementary Material 3. Fig. S3. FIP2 indirectly augments the interaction between N protein and RAB11A. (**A**) Specific interaction of RAB11A with N protein in vRNP. 293T cells were co-transfected with plasmids encoding N-Myc, HA-P, and Flag-RAB11A, either individually or jointly as indicated. At 36 h post transfection, cells were collected and subjected to co-immunoprecipitation assays. Proteins were immunoprecipitated using anti-Flag magnetic beads and analyzed by Western blot. (**B**) Interaction between FIP2 and N protein. 293T cells were transfected with the specified plasmids for 36 h. Protein interactions were assessed by immunoprecipitation using anti-Flag tag magnetic beads, followed by Western blot analysis.

Supplementary Material 4. Fig. S4. Cell penetration, cytotoxicity, and anti-HPIV3 activity of RBD in different cell lines. (**A**) Cellular uptake of FITC-labeled RBD in HeLa Cells. HeLa cells were exposed to 5 μM FITC-labeled RBD in the absence or presence of HPIV3_HA-P_ infection, and analyzed by fluorescent microscopy at 24 hpi. RBD (green), HPIV3_HA-P_ (red), and nuclei were stained with DAPI. Scale bar, 50 μm. (**B **to** F**) Cytotoxicity of RBD in various cell lines. Cell viability was assessed in HeLa (**B**), A549 (**C**), MK2 (**D**), MDCK (**E**) and HEp-2 cells (**F**) treated with increasing concentrations of YT-DRI for 24 h using the CCK-8 assay. (**G **to** I**) Antiviral efficacy of RBD against HPIV3 in different cell lines. A549 (**G**), MK2 (**H**) and MDCK (**I**) cells were treated with 2-fold-increasing concentrations of RBD for 24h, and viral titers in the cell supernatant were determined by TCID_50_. All experiments were independently repeated at least twice with consistent results. Data are means ± SEM.

Supplementary Material 5. Fig. S5. Cytotoxicity and antiviral activity of YT-DRI against HPIV3, RSV and IAV in different cell lines. (**A **to** D**) Cytotoxicity of YT-DRI in various cell lines. Cell viability was assessed in HeLa (**A**), A549 (**B**), MDCK (**C**) and HEp-2 cells (**D**) treated with increasing concentrations of YT-DRI for 24 h using the CCK-8 assay. (**E **and** F**) Antiviral potential of peptide YT-DRI against HPIV3. A549 (**E**) and MDCK cells (**F**), infected with HPIV3 (MOI=0.01), were treated with escalating concentrations of YT-DRI. At 24 hpi, TCID_50_ quantification revealed the inhibitory effects on viral titers. (**G **to** J**) Antiviral potential of peptide YT-DRI against RSV. A549 (**G**), HeLa (**H**), HEp-2 (**I**), and MDCK cells (**J**), infected with RSV (MOI=0. 1), were treated with escalating concentrations of YT-DRI. At 24 hpi, TCID_50_ quantification revealed the inhibitory effects on viral titers. (**K **to** M**) Inhibition of IAV infection by peptide YT-DRI. A549 (**K**), MDCK (**L**), and HeLa cells (**M**) were infected with IAV (MOI=0.01), followed by treatment with YT-DRI. At 24 hpi, TCID_50_ quantification confirmed the antiviral efficacy. (**N **to** O**) YT-DRI inhibits coinfection of HPIV3, RSV, and IAV in HeLa (**N**) and MDCK (**O**) cells. HeLa and MDCK cells, coinfected with HPIV3 (MOI=0.01), RSV (MOI=0. 1), and IAV (MOI=0.01), received 5 μM YT-DRI treatment. At 24 hpi, RT-qPCR analysis revealed suppressed viral RNA levels, underscoring the broad-spectrum antiviral activity. All experiments were independently replicated at least twice with consistent results. Statistical significance (*p < 0.05, **p < 0.01, ***p < 0.001, ****p < 0.0001 ) was determined by two-sided unpaired t-test. Data are presented as means ± SEM.

Supplementary Material 6. Fig. S6. YT-DRI treatment exerts RAB11A-dependent antiviral effect. (**A **and **B**) YT-DRI's antiviral efficacy relies on RAB11A. WT and RAB11A-depleted HeLa cells, infected with RSV (MOI of 0.1) or IAV (MOI of 0.01) and treated with YT-DRI (5 μM). The supernatants were assayed for viral titers at 24 hpi by TCID_50_. (**C**) Impact of RAB11A depletion on EV71 replication in HeLa cells. WT and RAB11A-depleted HeLa cells were infected with EV71 (MOI=0. 1) for 24 h, and viral titers in the cell supernatant were determined by TCID_50_. (**D**)The effects of increasing concentrations of YT-DRI on the replication of EV71. HeLa cells, infected with EV71(MOI=0. 1), received escalating concentrations of YT-DRI. At 24 hpi, TCID_50_ quantification revealed the inhibitory effects on viral titers. All experiments were independently replicated at least twice with consistent results. Statistical significance (*p < 0.05, **p < 0.01) was determined by two-sided unpaired t-test. Data are presented as means ± SEM.

Supplementary Material 7. Fig. S7. In Vivo biodistribution of Cy5-YT-DRI. (**A**) Mice were treated with PBS or Cy5-YT-DRI (5mg/kg) via intraperitoneal injection. Bioluminescence readings were conducted at different time points using the IVIS Lumina K Series III for ventral (the upper row) and dorsal (the lower row) views, and the average radiance is expressed as the sum of the photons per second from each pixel inside the region of interest per the number of pixels (p s−1 cm−2 sr−1). (**B-F**) Representative images of the brains(**B**), lungs (**C**), livers (**D**), kidneys (**E**), and hearts (**F**) from the PBS-treated or Cy5-YT-DRI-treated mice. (**G**) Normalized bioluminescence results are expressed as means ± SEM from at least two experiments. Statistical significance (*p < 0.05, **p < 0.01, ***P < 0.001) was determined by a two-sided unpaired t-test. Data are presented as means ± SEM.

Supplementary Material 8. Fig. S8. In vivo safety of YT-DRI. (**A**) Body weight changes of the 6-week-old ICR mice treated with PBS or 5 mg/kg or 10 mg/kg of YT-DRI at the indicated time points. (**B**) Hematoxylin and eosin staining of various tissues under an optical microscope for histopathological morphology analysis at 14 days after i.p. administration. Scale bar, 200 μm.

Supplementary Material 9.

## Data Availability

All study data are included in the article and SI Appendix.
