# Supplementary figures and images for: Peptides targeting RAB11A–FIP2 complex inhibit HPIV3, RSV, and IAV replication as broad-spectrum antivirals
Source: Cell Biosci. 2025 Apr 21;15:50. doi: 10.1186/s13578-025-01384-z (PMC12013085; doi:10.1186/s13578-025-01384-z)

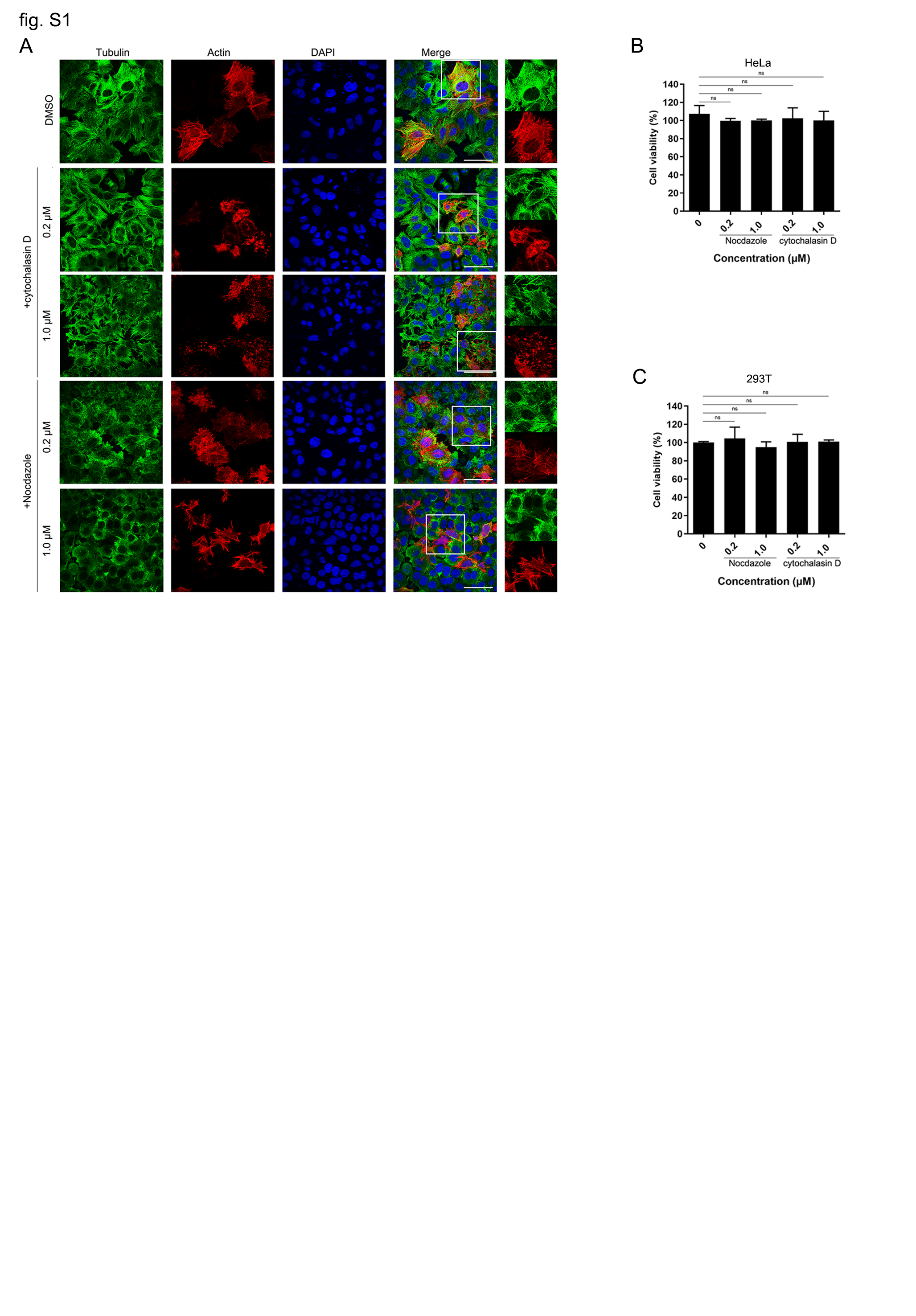

Supplement: Supplementary file 1 — Supplementary Material 1. Fig. S1. The specific effects and cytotoxicity of Nocodazole and Cytochalasin D. (A) Hela cells were transfected with mCherry-Lifeact-expressing plasmids. After 24 h, cells were treated with indicated concentrations of Nocodazole or Cytochalasin D for 6 h. The resulting images, captured through fluorescent microscopy, depict F-actin labeled with mCherry-Lifeact (red), microtubules stained using a mouse anti-α-tubulin primary antibody (green), and nuclei counterstained with DAPI (blue). Scale bar, 50 μm. Cell viability was assessed in HeLa (B) and 293T (C) cells treated with indicated concentrations of Nocodazole or Cytochalasin D for 6 h using the CCK-8 assay. All experiments were independently repeated at least twice with reproducible results. Statistical significance (ns, p > 0.05) was determined by two-sided unpaired t-test for statistical significance and log-rank test for survival curves. Data are presented as means ± SEM. [file 13578_2025_1384_MOESM1_ESM.tif]

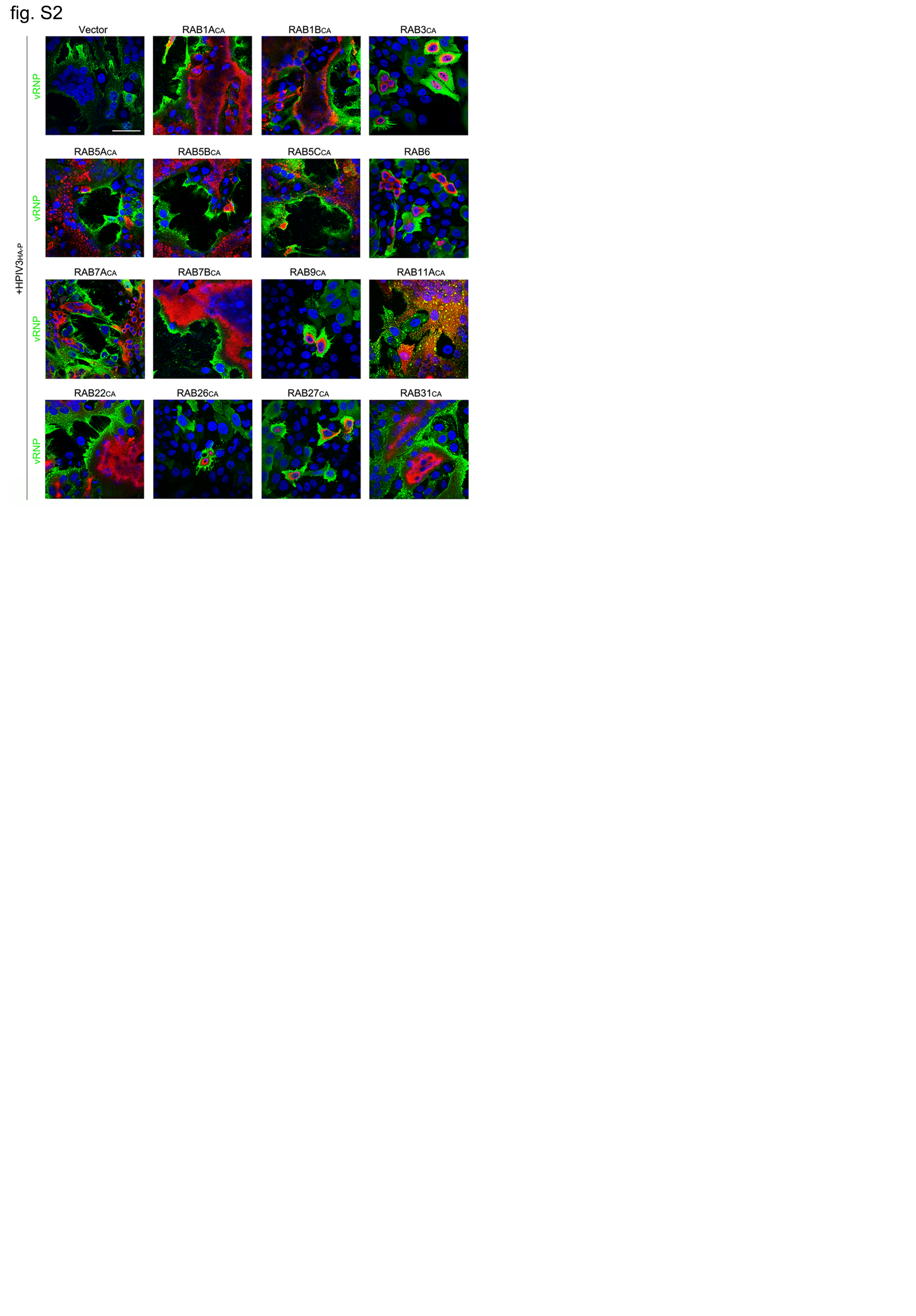

Supplement: Supplementary file 2 — Supplementary Material 2. Fig. S2. The specific co-localization of RAB11ACA and vRNP. HeLa cells were transfected with plasmids expressing the specified constitutively active RAB GTPases tagged with Flag, and then subsequently infected with HPIV3HA-P (MOI=0.01) for 24 h. Immunofluorescent staining was conducted using rabbit anti-HA antibody (in red) and mouse anti-Flag antibody (in green), while nuclei were counterstained with DAPI. Scale bar, 50 μm. [file 13578_2025_1384_MOESM2_ESM.tif]

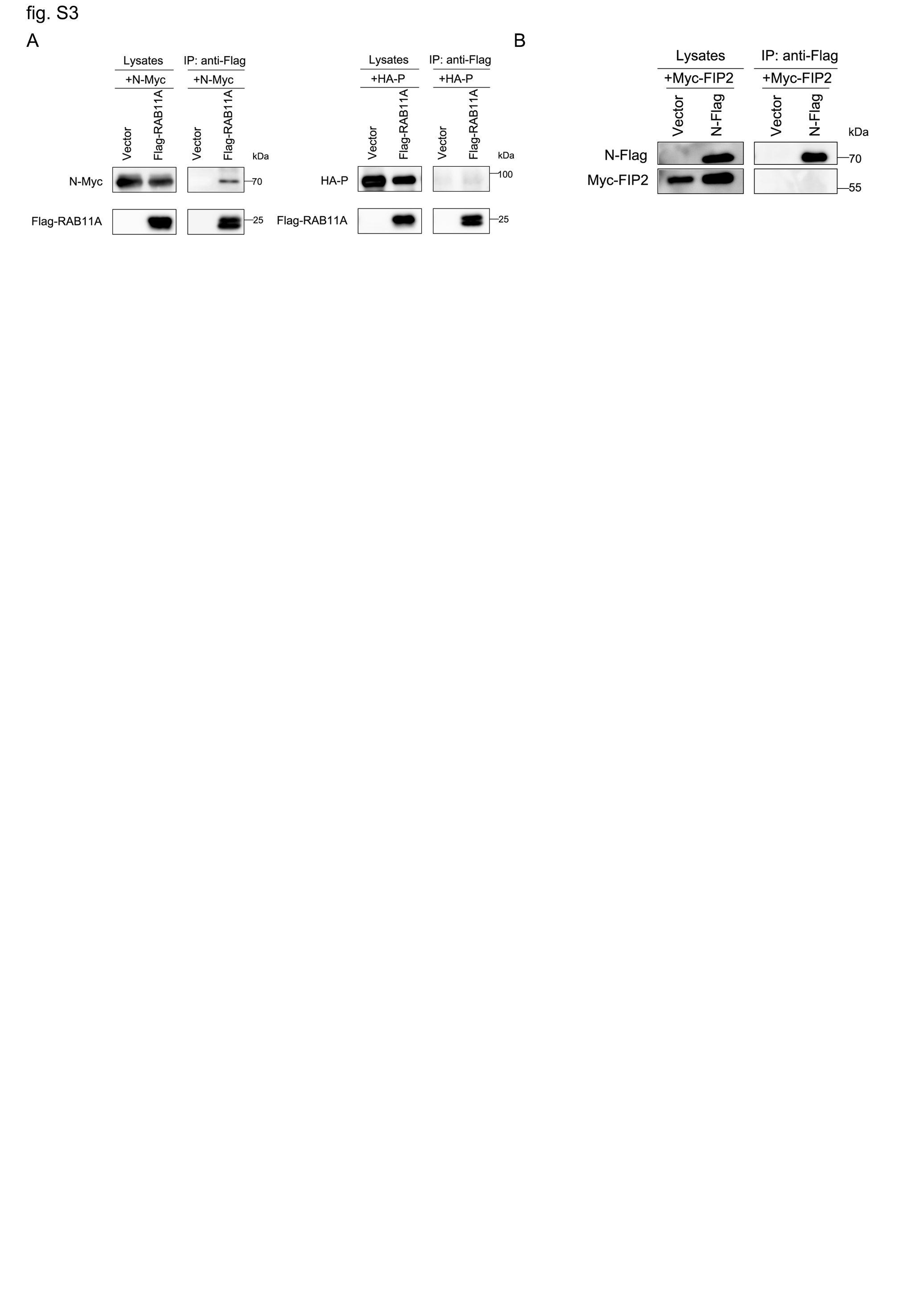

Supplement: Supplementary file 3 — Supplementary Material 3. Fig. S3. FIP2 indirectly augments the interaction between N protein and RAB11A. (A) Specific interaction of RAB11A with N protein in vRNP. 293T cells were co-transfected with plasmids encoding N-Myc, HA-P, and Flag-RAB11A, either individually or jointly as indicated. At 36 h post transfection, cells were collected and subjected to co-immunoprecipitation assays. Proteins were immunoprecipitated using anti-Flag magnetic beads and analyzed by Western blot. (B) Interaction between FIP2 and N protein. 293T cells were transfected with the specified plasmids for 36 h. Protein interactions were assessed by immunoprecipitation using anti-Flag tag magnetic beads, followed by Western blot analysis. [file 13578_2025_1384_MOESM3_ESM.tif]

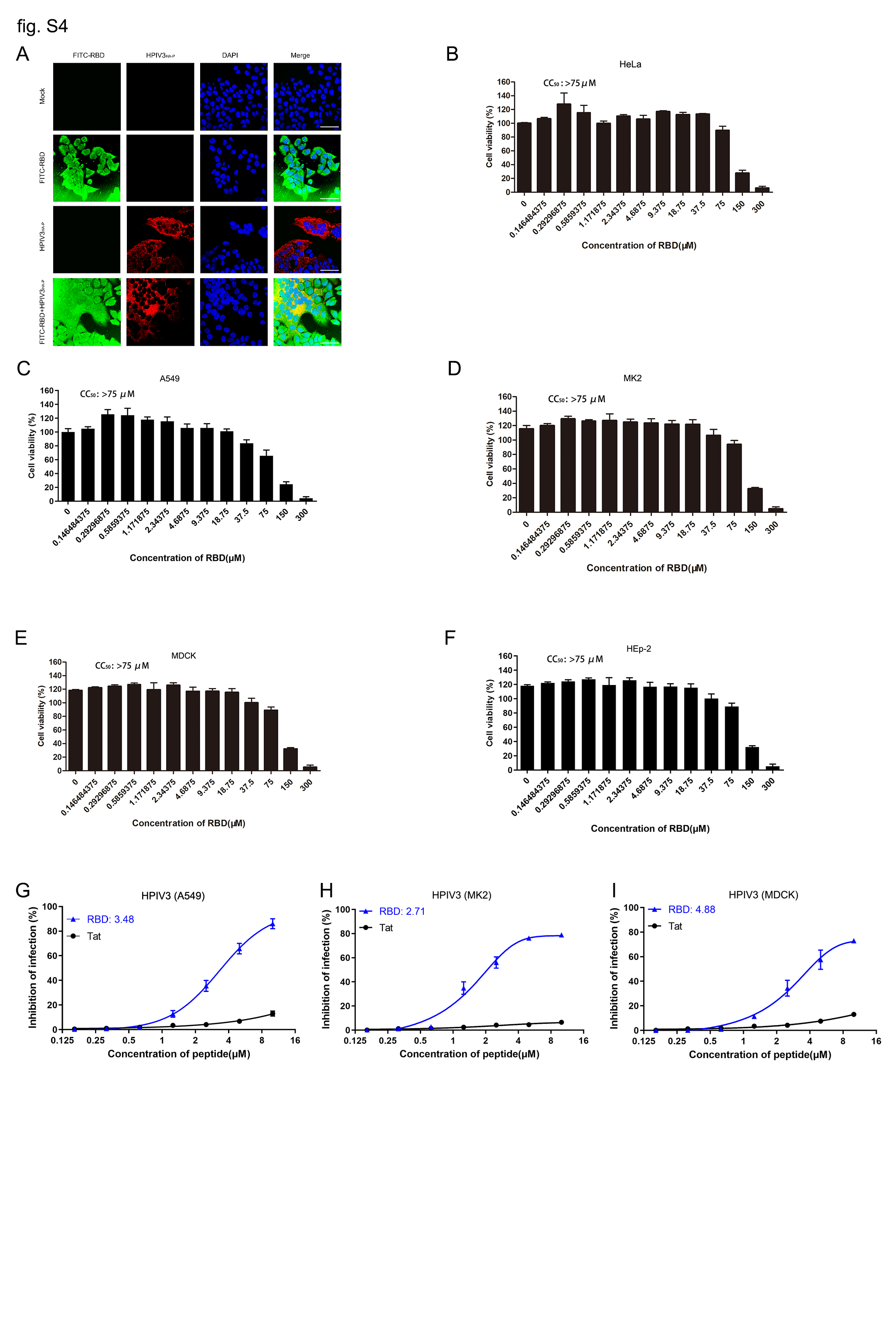

Supplement: Supplementary file 4 — Supplementary Material 4. Fig. S4. Cell penetration, cytotoxicity, and anti-HPIV3 activity of RBD in different cell lines. (A) Cellular uptake of FITC-labeled RBD in HeLa Cells. HeLa cells were exposed to 5 μM FITC-labeled RBD in the absence or presence of HPIV3HA-P infection, and analyzed by fluorescent microscopy at 24 hpi. RBD (green), HPIV3HA-P (red), and nuclei were stained with DAPI. Scale bar, 50 μm. (B to F) Cytotoxicity of RBD in various cell lines. Cell viability was assessed in HeLa (B), A549 (C), MK2 (D), MDCK (E) and HEp-2 cells (F) treated with increasing concentrations of YT-DRI for 24 h using the CCK-8 assay. (G to I) Antiviral efficacy of RBD against HPIV3 in different cell lines. A549 (G), MK2 (H) and MDCK (I) cells were treated with 2-fold-increasing concentrations of RBD for 24h, and viral titers in the cell supernatant were determined by TCID50. All experiments were independently repeated at least twice with consistent results. Data are means ± SEM. [file 13578_2025_1384_MOESM4_ESM.tif]

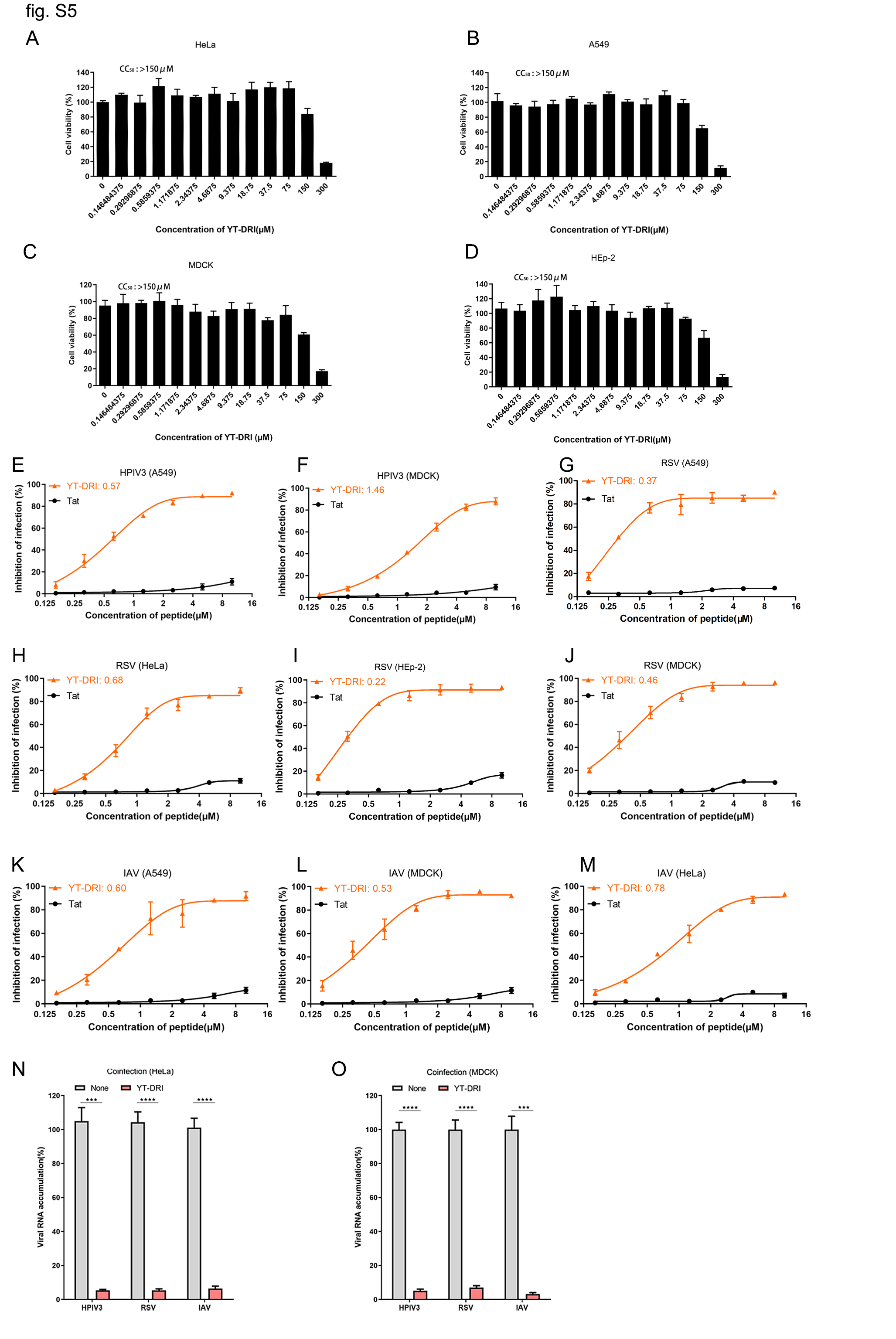

Supplement: Supplementary file 5 — Supplementary Material 5. Fig. S5. Cytotoxicity and antiviral activity of YT-DRI against HPIV3, RSV and IAV in different cell lines. (A to D) Cytotoxicity of YT-DRI in various cell lines. Cell viability was assessed in HeLa (A), A549 (B), MDCK (C) and HEp-2 cells (D) treated with increasing concentrations of YT-DRI for 24 h using the CCK-8 assay. (E and F) Antiviral potential of peptide YT-DRI against HPIV3. A549 (E) and MDCK cells (F), infected with HPIV3 (MOI=0.01), were treated with escalating concentrations of YT-DRI. At 24 hpi, TCID50 quantification revealed the inhibitory effects on viral titers. (G to J) Antiviral potential of peptide YT-DRI against RSV. A549 (G), HeLa (H), HEp-2 (I), and MDCK cells (J), infected with RSV (MOI=0. 1), were treated with escalating concentrations of YT-DRI. At 24 hpi, TCID50 quantification revealed the inhibitory effects on viral titers. (K to M) Inhibition of IAV infection by peptide YT-DRI. A549 (K), MDCK (L), and HeLa cells (M) were infected with IAV (MOI=0.01), followed by treatment with YT-DRI. At 24 hpi, TCID50 quantification confirmed the antiviral efficacy. (N to O) YT-DRI inhibits coinfection of HPIV3, RSV, and IAV in HeLa (N) and MDCK (O) cells. HeLa and MDCK cells, coinfected with HPIV3 (MOI=0.01), RSV (MOI=0. 1), and IAV (MOI=0.01), received 5 μM YT-DRI treatment. At 24 hpi, RT-qPCR analysis revealed suppressed viral RNA levels, underscoring the broad-spectrum antiviral activity. All experiments were independently replicated at least twice with consistent results. Statistical significance (*p < 0.05, **p < 0.01, ***p < 0.001, ****p < 0.0001 ) was determined by two-sided unpaired t-test. Data are presented as means ± SEM. [file 13578_2025_1384_MOESM5_ESM.tif]

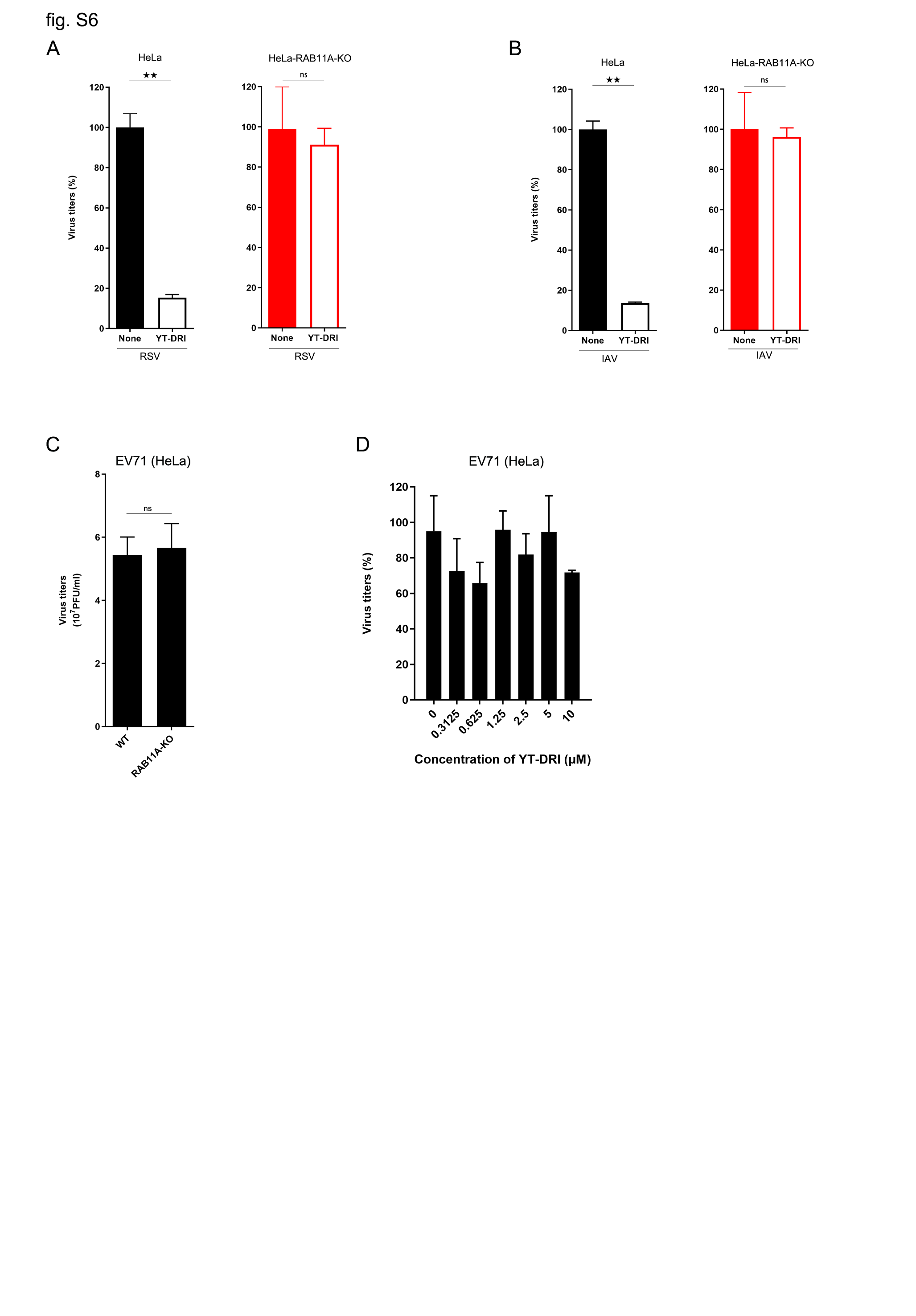

Supplement: Supplementary file 6 — Supplementary Material 6. Fig. S6. YT-DRI treatment exerts RAB11A-dependent antiviral effect. (A and B) YT-DRI's antiviral efficacy relies on RAB11A. WT and RAB11A-depleted HeLa cells, infected with RSV (MOI of 0.1) or IAV (MOI of 0.01) and treated with YT-DRI (5 μM). The supernatants were assayed for viral titers at 24 hpi by TCID50. (C) Impact of RAB11A depletion on EV71 replication in HeLa cells. WT and RAB11A-depleted HeLa cells were infected with EV71 (MOI=0. 1) for 24 h, and viral titers in the cell supernatant were determined by TCID50. (D)The effects of increasing concentrations of YT-DRI on the replication of EV71. HeLa cells, infected with EV71(MOI=0. 1), received escalating concentrations of YT-DRI. At 24 hpi, TCID50 quantification revealed the inhibitory effects on viral titers. All experiments were independently replicated at least twice with consistent results. Statistical significance (*p < 0.05, **p < 0.01) was determined by two-sided unpaired t-test. Data are presented as means ± SEM. [file 13578_2025_1384_MOESM6_ESM.tif]

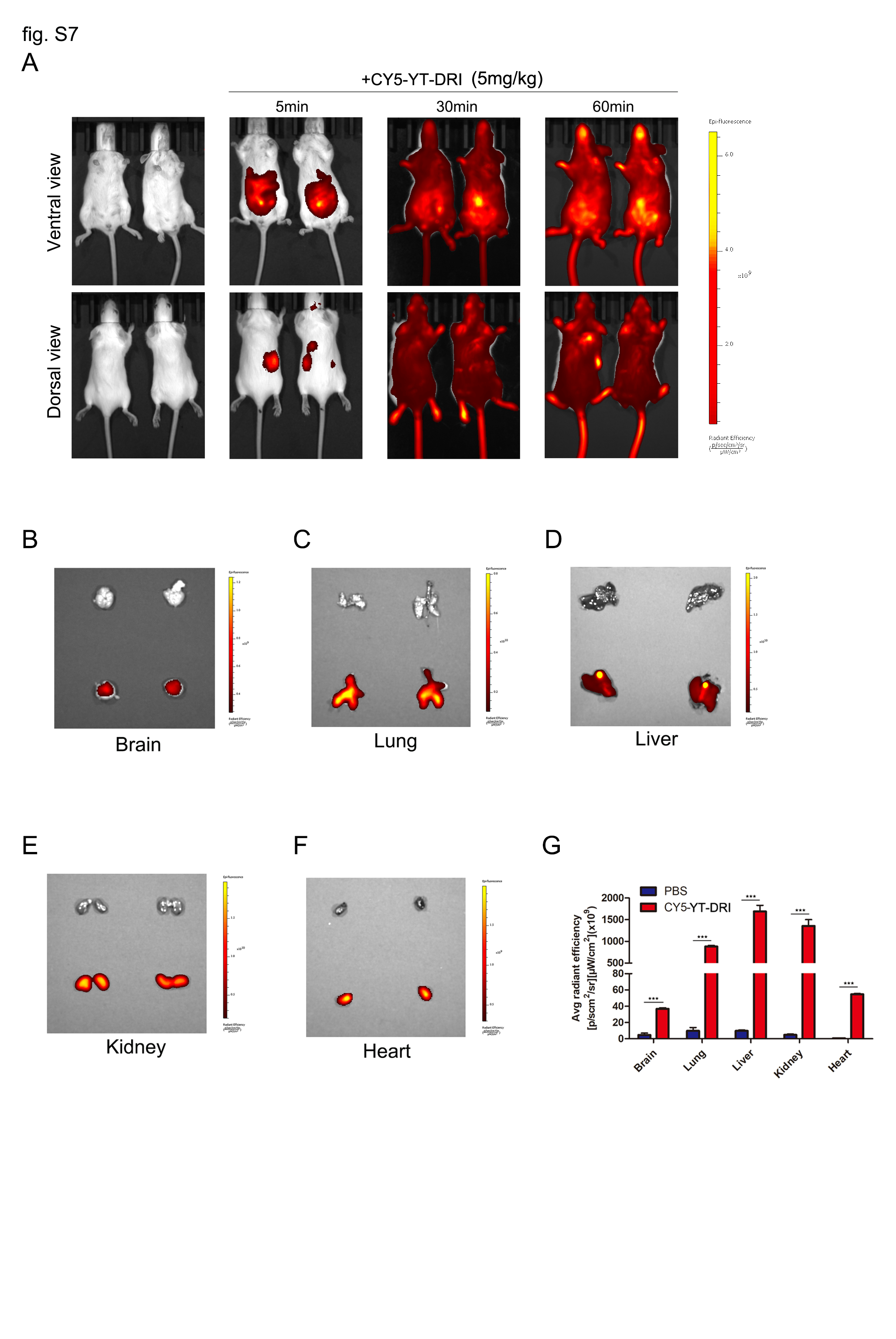

Supplement: Supplementary file 7 — Supplementary Material 7. Fig. S7. In Vivo biodistribution of Cy5-YT-DRI. (A) Mice were treated with PBS or Cy5-YT-DRI (5mg/kg) via intraperitoneal injection. Bioluminescence readings were conducted at different time points using the IVIS Lumina K Series III for ventral (the upper row) and dorsal (the lower row) views, and the average radiance is expressed as the sum of the photons per second from each pixel inside the region of interest per the number of pixels (p s−1 cm−2 sr−1). (B-F) Representative images of the brains(B), lungs (C), livers (D), kidneys (E), and hearts (F) from the PBS-treated or Cy5-YT-DRI-treated mice. (G) Normalized bioluminescence results are expressed as means ± SEM from at least two experiments. Statistical significance (*p < 0.05, **p < 0.01, ***P < 0.001) was determined by a two-sided unpaired t-test. Data are presented as means ± SEM. [file 13578_2025_1384_MOESM7_ESM.tif]

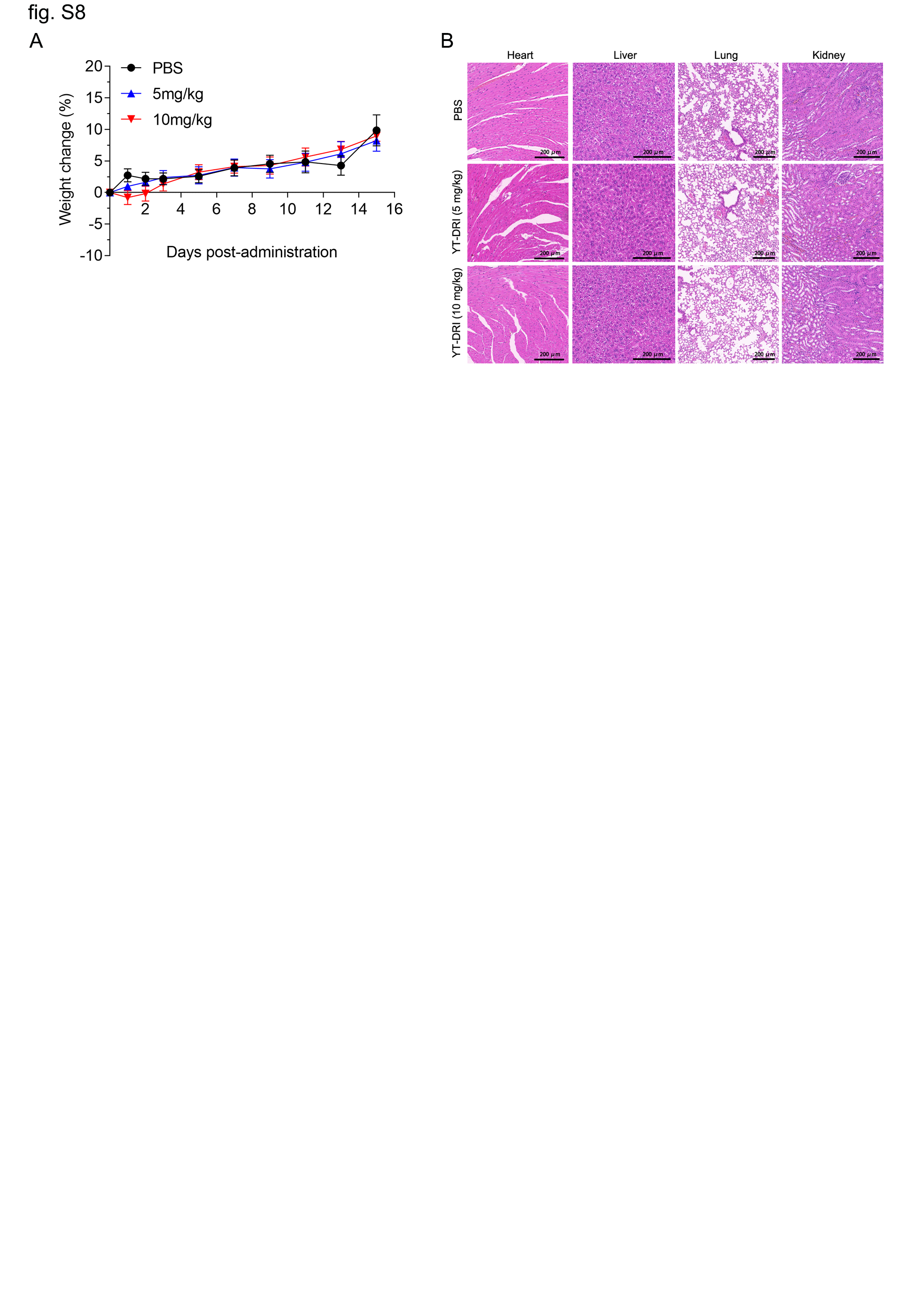

Supplement: Supplementary file 8 — Supplementary Material 8. Fig. S8. In vivo safety of YT-DRI. (A) Body weight changes of the 6-week-old ICR mice treated with PBS or 5 mg/kg or 10 mg/kg of YT-DRI at the indicated time points. (B) Hematoxylin and eosin staining of various tissues under an optical microscope for histopathological morphology analysis at 14 days after i.p. administration. Scale bar, 200 μm. [file 13578_2025_1384_MOESM8_ESM.tif]
