# Supplementary material for: Peptides targeting RAB11A–FIP2 complex inhibit HPIV3, RSV, and IAV replication as broad-spectrum antivirals
Source: Cell Biosci. 2025 Apr 21;15:50. doi: 10.1186/s13578-025-01384-z (PMC12013085; doi:10.1186/s13578-025-01384-z)
Supplement: Supplementary file 9 — Supplementary Material 9. [file 13578_2025_1384_MOESM9_ESM.docx]

**Table S1. The primers used in this study**

| Primers | Sequence (5' to 3') | Purpose |
| --- | --- | --- |
| sg-RAB11A-1-PF | CACCGCATTTCGAGTAAATCGAGAC | sgRNAs |
| sg-RAB11A-1-PR | AAACGTCTCGATTTACTCGAAATGC |  |
| sg-RAB11A-2-PF | CACCGTTCGCTCCTCGGCCGCGCAA |  |
| sg-RAB11A-2-PR | AAACTTGCGCGGCCGAGGAGCGAAC |  |
| sg-RAB11FIP2-1-PF | CACCGGCTACCTGGATTGCTAATTC |  |
| sg-RAB11FIP2-1-PR | AAACGAATTAGCAATCCAGGTAGCC |  |
| sg-RAB11FIP2-2-PF | CACCGCGTGGGTTGGAAACCACTTT |  |
| sg-RAB11FIP2-2-PR | AAACAAAGTGGTTTCCAACCCACGC |  |
| IAV-PF | GACTCACATGATGATCTGGCA | qRT-PCR |
| IAV-PR | CTTGTTCTCCGTCCATTCTCA |  |
| RSV-PF | CGAGCCAGAAGAGAACTACCA |  |
| RSV-PR | CCTTCTAGGTGCAGGACCTTA |  |
| HPIV3-PF | GCCCTTGGACCGACAATAAC |  |
| HPIV3-PR | GCTCTGGATTGGCATAAGCC |  |
| GAPDH-PF | AAGGCTGTGGGCAAGG |  |
| GAPDH-PR | TGGAGGAGTGGGTGTCG |  |
